# A robust clustering strategy for stratification unveils unique patient subgroups in acutely decompensated cirrhosis

**DOI:** 10.1186/s12967-024-05386-2

**Published:** 2024-06-27

**Authors:** Sara Palomino-Echeverria, Estefania Huergo, Asier Ortega-Legarreta, Eva M. Uson Raposo, Ferran Aguilar, Carlos de la Peña-Ramirez, Cristina López-Vicario, Carlo Alessandria, Wim Laleman, Alberto Queiroz Farias, Richard Moreau, Javier Fernandez, Vicente Arroyo, Paolo Caraceni, Vincenzo Lagani, Cristina Sánchez-Garrido, Joan Clària, Jesper Tegner, Jonel Trebicka, Narsis A. Kiani, Nuria Planell, Pierre-Emmanuel Rautou, David Gomez-Cabrero

**Affiliations:** 1 Unit of Translational Bioinformatics, Navarrabiomed - Fundación Miguel Servet, Pamplona, Spain; 2 European Foundation for the Study of Chronic Liver Failure, Barcelona, Spain; 3 Biochemistry and Molecular Genetics Service, Hospital Clínic-IDIBAPS, Barcelona, Spain; 4 CIBERehd, Barcelona, Spain; 5 Department of Biomedical Sciences, University of Barcelona, Barcelona, Spain; 6 Division of Gastroenterology and Hepatology, A.O.U. Città della Salute e della Scienza di Torino, Torino, Italy; 7 Department of Gastroenterology & Hepatology, Section of Liver & Biliopancreatic disorders and Liver Transplantation, University Hospitals Leuven, KU LEUVEN, Leuven, Belgium; 8 Department of Gastroenterology, Hospital das Clínicas, University of São Paulo School of Medicine, Paulo School, Brazil; 9 Université Paris-Cité, Inserm, Centre de recherche sur l’inflammation, UMR 1149, Paris, France; 10 Assistance Publique-Hôpitaux de Paris (AP-HP), Paris, France; 11 Hôpital Beaujon, Service d‘Hépatologie, Clichy, France; 12 Department of Medical and Surgical Science, University of Bologna, Bologna, Italy; 13 Biological and Environmental Science and Engineering Division, King Abdullah University of Science and Technology (KAUST), Thuwal, Saudi Arabia; 14 SDAIA-KAUST Center of Excellence in Data Science and Artificial Intelligence, Thuwal, Saudi Arabia; 15 Institute of Chemical Biology, Ilia State University, Tbilisi, 0162 Georgia; 16 Department of internal medicine B, University of Münster, Münster, Germany; 17 Algorithmic Dynamics Lab, Center for Molecular Medicine, Karolinska Institutet, Solna, Sweden; 18 Computational Biology Program, Universidad de Navarra, CIMA, Instituto de Investigación Sanitaria de Navarra (IdiSNA), Navarra, 31008 Spain; 19 AP-HP, Hôpital Beaujon, Service d’Hépatologie, DMU DIGEST, Centre de Référence des Maladies Vasculaires du Foie, FILFOIE, ERN RARE-LIVER, Clichy, France; 20 Department of Oncology-Pathology, Karolinska Institutet, Solna, Sweden; 21 IRCCS Azienda Ospedaliera-Universitaria di Bologna, Bologna, Italy; 22 Unit of Computational Medicine, Department of Medicine, Center for Molecular Medicine, Karolinska Institutet, Karolinska University Hospital, Stockholm, Sweden; 23 Computer, Electrical and Mathematical Sciences and Engineering Division, King Abdullah University of Science and Technology (KAUST), Thuwal, Saudi Arabia

**Keywords:** Stratification, Clustering, Complex diseases, Cirrhosis, ACLF, Patient heterogeneity, Unsupervised learning

## Abstract

**Background:**

Patient heterogeneity poses significant challenges for managing individuals and designing clinical trials, especially in complex diseases. Existing classifications rely on outcome-predicting scores, potentially overlooking crucial elements contributing to heterogeneity without necessarily impacting prognosis.

**Methods:**

To address patient heterogeneity, we developed ClustALL, a computational pipeline that simultaneously faces diverse clinical data challenges like mixed types, missing values, and collinearity. ClustALL enables the unsupervised identification of patient stratifications while filtering for stratifications that are robust against minor variations in the population (population-based) and against limited adjustments in the algorithm’s parameters (parameter-based).

**Results:**

Applied to a European cohort of patients with acutely decompensated cirrhosis (*n* = 766), ClustALL identified five robust stratifications, using only data at hospital admission. All stratifications included markers of impaired liver function and number of organ dysfunction or failure, and most included precipitating events. When focusing on one of these stratifications, patients were categorized into three clusters characterized by typical clinical features; notably, the 3-cluster stratification showed a prognostic value. Re-assessment of patient stratification during follow-up delineated patients’ outcomes, with further improvement of the prognostic value of the stratification. We validated these findings in an independent prospective multicentre cohort of patients from Latin America (*n* = 580).

**Conclusions:**

By applying ClustALL to patients with acutely decompensated cirrhosis, we identified three patient clusters. Following these clusters over time offers insights that could guide future clinical trial design. ClustALL is a novel and robust stratification method capable of addressing the multiple challenges of patient stratification in most complex diseases.

**Supplementary Information:**

The online version contains supplementary material available at 10.1186/s12967-024-05386-2.

## Background

Heterogeneity is a prevalent phenomenon observed in numerous diseases, including various types of cancer [1], autoimmune conditions like multiple sclerosis [2], and diabetes [3]. Substantial interindividual changes in phenotype and pathophysiology within a disease often limit the effectiveness of traditional “one-size-fits-all” medicine approaches. This becomes especially critical in diseases where environmental and lifestyle factors also play a significant role. Acutely decompensated cirrhosis, which refers to the rapid development of overt ascites, overt hepatic encephalopathy, variceal haemorrhage, or any combination of these disorders, which often leads to nonelective admission to the hospital of patients who were previously stable, exemplifies significant inter-individual variability [4, 5]. Acutely decompensated cirrhosis encompasses a range of causes of cirrhosis, including alcohol consumption, metabolic dysfunction, viral hepatitis, genetic disorders, or autoimmune biliary diseases. It is often accompanied by comorbidities, which are neither causes nor consequences of cirrhosis, but they increase mortality [5, 6]. Heterogeneity of cirrhosis can also include various precipitating events such as infection or alcoholic-related hepatitis, diverse clinical presentations like ascites, gastrointestinal bleeding, and hepatic encephalopathy, and multiple possible outcomes such as cancer, liver failure, or death. This clinical heterogeneity poses a considerable challenge as it likely accounts for the diverse responses to treatment and outcomes observed in these patients [7]. Therefore, we reasoned that analysing a large population of patients with acutely decompensated cirrhosis should allow us to develop stratification tools.

A major tool for the characterization of patient heterogeneity is the identification of patient subtypes, also defined as patient stratification. Importantly, the World Health Organization (WHO) has acknowledged patient stratification as a valuable approach for enhancing population health management and providing better-tailored services [8]. In conceptual terms, patient stratification can be described as the process of grouping or clustering patients based on specific characteristics or patterns without relying on labelled data or information about future outcomes [9]. Therefore, contrary to scores developed using classical statistical approaches based on the clinical course, stratification can capture features explaining patients’ heterogeneity independently of their association with patient outcomes.

Numerous attempts have been made to identify subgroups within clinical datasets [9–11]. However, the lack of a universally applicable approach poses a significant challenge in the field of clustering analysis. Although there have been advancements beyond the classical k-means and hierarchical clustering methods, no general framework still allows the organization and classification of clustering methodologies in the clinical setting [12]. Instead, many ad-hoc applications have been developed for specific scenarios, but their generalizability is often limited. Horne et al.‘s review highlights how certain disease labels, such as asthma, can encompass diverse symptoms and causes [10]. They illustrate this lack of generalization by noting that they found 63 studies utilizing cluster analysis to identify different asthma subtypes based on various clinical data. While there is no global classification, these applications can be grouped based on specific characteristics such as managing missing values, collinearity, or mixed data [11]. For instance, when handling missing data, some methods exclude samples from the analysis, potentially resulting in a loss of statistical power, while others rely on a single imputation, overlooking the potential bias that can be introduced [13]. Highly correlated variables represent a challenge. Some methods exclude them, while others employ dimensionality reduction techniques such as Principal Component (PC) reduction to capture underlying lower-dimensional data patterns [14, 15]. However, both decisions may affect the outcome of the clustering, as sensitivity analyses are rarely conducted. Moreover, indiscriminate feature selection can inadvertently remove informative features along with noisy ones, potentially biasing the results [16]. Furthermore, most clustering methodologies assume the existence of a single stratification, disregarding the possibility of having none or multiple valid alternatives for subgrouping the population [17]. Interestingly, trace-based clustering methodologies have recently emerged to aid in the interpretation and validation of the identified subgroups, often requiring domain knowledge and expert input [18]. Within this technique, the proposal involves tracking elements across clusters generated by different runs of the clustering algorithm to identify stable and informative patterns in the data set.

Additionally, the evaluation of clustering outcomes is an open problem that is based on the quality of the produced clusters. In the case of unsupervised clustering, where no preliminary classification exists, evaluations are typically referenced against theoretical benchmarks. For instance, when addressing the optimal number of clusters, various quality metrics are available, such as the clustering coefficient [19] or the silhouette index [20], among many others. Importantly, while there is no universal methodology that excels across all scenarios for all data sets, as dictated by the “*no free lunch*” theorem [21], there exist strategies that yield high-quality results [22–24]. Another essential measure—referred to as robustness—lacks a precise definition. Robustness, in general terms, signifies the capacity of a system to withstand changes [25]. In our context, we investigate whether a clustering remains stable when subjected to perturbations. In this work, we considered two types of perturbations: those derived from changes in the population and those arising from changes in the algorithm’s parameters. In the case of population-based perturbations, we quantify how a given clustering is influenced by variations in the underlying population. Bootstrapping is one approach to address this scenario [26]. In the case of parameter-based perturbations, we assess the impact of parameter adjustments in the clustering algorithm on the identified clustering [27]. Consider a scenario where a parameter “x” defines our clustering strategy. How different is the resulting clustering when using “x = 1” versus “x = 1.1”? Here, robustness translates to clusterings that maintain stability even when parameter values shift. For the reader’s clarity, we will name the two different robustness criteria: **population-based robustness** and **parameter-based robustness.**

Importantly, there is currently no methodology capable of addressing all the aforementioned scenarios while ensuring both definitions of robustness. To address these challenges comprehensively, we developed ClustALL, a novel framework that robustly identifies patient subgroups by addressing all the previously mentioned challenges and limitations of existing methodologies. We applied ClustALL -as a proof-of-concept- to a large prospective cohort of patients non-electively admitted to the hospital for acutely decompensated cirrhosis.

In this study, ClustALL addressed the stratification challenge within a dataset of patients with acutely decompensated cirrhosis [28], characterized by the presence of missing data, mixed data types, and correlated features. It revealed multiple stratification solutions, with one exhibiting special interest in the clinical context and showing prognostic value. We then validated the reproducibility of this stratification using a separate prospective cohort of patients, affirming ClustALL’s robustness and reliability. One further aim of the study was to demonstrate the usability of stratification over the disease course, showcasing its prognostic value. Looking beyond cirrhosis, ClustALL holds promise for broader applications in diverse clinical settings, suggesting its potential to revolutionize patient subgroup identification and improve healthcare management.

## Methods

### ClustALL framework

Given a set of patients affected by a complex disease with clinical data available, the goal of ClustALL is to identify all the possible alternatives to stratify them that are robust and consistent, even when different parameters or settings are used to generate the stratifications (distance metric, clustering algorithm, and the number of imputations).

The Supplementary Methods include a glossary of technical terms with explanations to elucidate technical terminology.

### Input data

ClustALL accepts binary, categorical, and numerical clinical variables as input (e.g., biochemical markers, demographics, clinical scores). Categorical features are transformed internally using a one-hot encoder method, avoiding the assumption of ordinal relationships between categories, which is essential for many clustering algorithms to operate efficiently. A minimum of two features is required, but including more features would lead to more precise clustering. It is important to note that increasing the number of features may also increase the computation time.

#### Step 1. Data complexity reduction

In this step, highly correlated features are replaced by a reduced set of variables that account for their variability. To that end:

**Step 1.1. Dendrogram** Hierarchical clustering is applied to the complete dataset, resulting in a dendrogram where variables are grouped based on similarity or collinearity [29]. The depth of each branch represents the distance between the groups of variables. All the possible depths of the dendrogram are extracted, and the sets of variables beneath each depth are stored as *Depth* (see *Glossary of Technical Terms, Supplementary Methods)*.

**Step 1.2. Preprocessing** Principal Component Analysis (PCA) is computed for each set of variables corresponding to each *Depth*, and the first three principal components are stored in a new matrix (*Embedding*) (see *Glossary of Technical Terms, Supplementary Methods)* [30]. For sets that contain only one variable, the variable itself is stored to generate the replacement matrix. This results in a complexity-reduced data set (*Embedding*) for each considered *Depth*. A subset of depths can be considered when the number of variables is too large.

#### Step 2. Stratification process

In this step, ClustALL calculates and pre-evaluates stratifications for each *Embedding*. For each *Embedding*, the dissimilarity between patients’ pairs is computed using correlation-based distance and Gower dissimilarity metric, resulting in two distance matrices [31, 32]. Clustering algorithms are then applied [33–35] depending on the distance used: k-means and hierarchical clustering for correlation distance matrices, and k-medoids and hierarchical clustering for the Gower distance matrix. Throughout all experiments, five different cluster numbers are evaluated k$?\left\{2, 3, 4, 5, 6\right\}$. The optimal number of clusters for each strategy is determined based on the consensus from three different measures of clustering internal validation: the sum-of-squares based index or WB-ratio, the Dunn index, and the average silhouette width [36, 37]. The objective is to group patients with comparable data while ensuring that patients in separate clusters are as dissimilar as possible from those in other clusters. As the output for this step, a stratification is derived for each combination denoted as “*embedding + distance metric + clustering method*”.

#### Step 3. Consensus-based stratifications

**Step 3.1. Population-based robustness** A data-driven threshold is used to define population-based robust subgroups or clusters. For each resulting stratification from the previous step, cluster-wise stability is computed by bootstrapping the dataset 1,000 times and calculating the Jaccard similarity index to the originally defined clusters (see *Glossary of Technical Terms, Supplementary Methods)* [38, 39]. Based on data distribution, stratifications with less than 85% stability (Fig. S4) are excluded. The remaining stratifications are denoted as *Strat*_filt_.

**Fig. 1:**
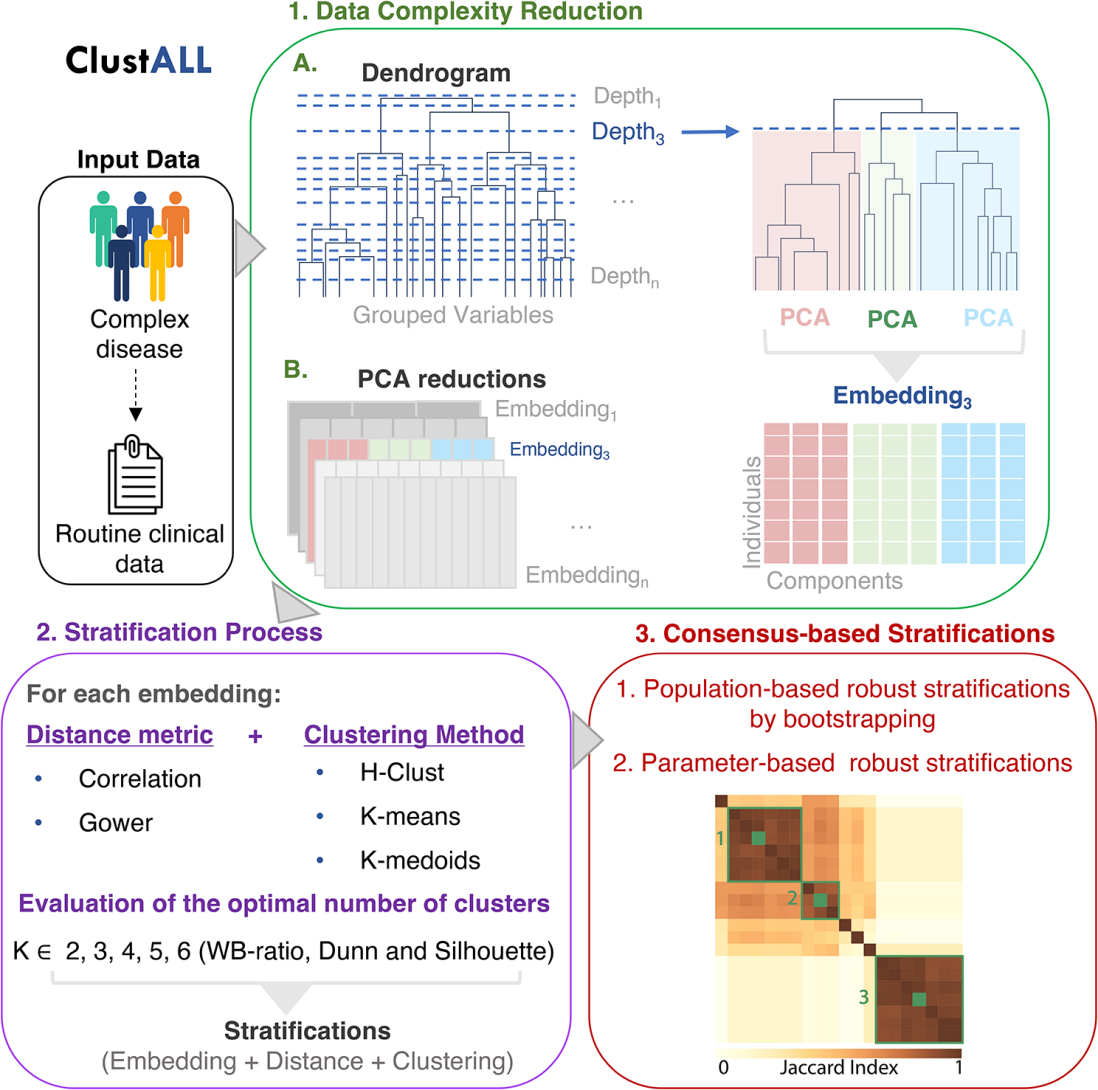
Schematic overview of the different steps of ClustALL approach (best viewed in colour). ClustALL takes clinical variables as input. First, data complexity is reduced by grouping the features into a dendrogram, assessing the resulting depths, and using Principal Component Analysis (PCA) (green panel). The output is an embedding for each possible depth. Then, stratification is computed considering the combination of different distance measures, clustering techniques, and cluster numbers (K) (purple panel). In the final step, non-robust stratifications are filtered, and the centroids derived from computing Jaccard (coloured green squares) similarity among the robust stratifications (green squares) are considered the final representatives of the stratifications (red panel)

**Step 3.2. Jaccard distance** is applied to compute distances between the population-based robust stratifications [38]. Then, to identify parameter-based robust clusters (where a minor modification in parameter selection provides a similar result), ClustALL considers those combinations that are part of a group of stratifications (green squares in Consensus-based stratifications step in Fig. 1). Then, as initial criteria, that can be modified by the user, centroids from each “combination group” are selected as parameter-based robust stratifications (coloured green squares in Consensus-based stratifications step in Fig. 1). The outcome can be none, one, or multiple ways to stratify the population robustly. In the current analysis, we considered parameter-based robust representatives: centroids of a combination group that includes at least 5 population-based robust stratifications.

### ClustALL enables input data with missing values

ClustALL can be adapted to work with missing data (Fig. S1). To that end, the ClustALL method is modified as follows:

#### Step 1 Adaptation

First, a dendrogram and its associated depths are computed considering the original dataset with missing values. The original dataset is then imputed 1,000 times using the Multivariate Imputation by Chained Equations (MICE) algorithm [40]. MICE is chosen for its capability to manage complex data structures and capture relationships between variables more effectively than other imputation methods [40]. Moreover, MICE offers the flexibility to specify relationships between variables through the *predictorMatrix* parameter within the *mice* function. This parameter allows us to handle interdependencies among input variables by specifying predictors for each target feature, thereby facilitating robust imputation.

Additionally, we employ the most suitable imputation method included in the *mice* function based on the data type. Specifically, predictive mean matching (*pmm*) is utilized for numeric variables, logistic regression (*logreg*) for factor variables with two levels, multinomial logit model (*polyreg*) for factor variables with more than two levels, and ordered logit model (*polr*) for ordered variables with multiple levels. Note that, by implementing imputation iteratively, we mitigate bias by capturing the inherent uncertainty and variability in the process. Subsequently, for each previously calculated *Depth* and each imputed dataset, the Data Complexity Reduction step is applied.

#### Step 2 Adaptation

Step 2.1 is computed for each combination of depth, distance metric, clustering algorithm and each *Embedding* derived from an imputed dataset. The selection of the optimal number of clusters is based on the consensus from cluster internal validation and the mode of the imputed datasets for each corresponding embedding. Afterwards, a distance matrix (*D*_*mat*_) between individuals is obtained by computing how often two individuals are assigned to the same cluster in each imputation (Fig. S1). Then, *D*_*mat*_ calculates a final stratification score using correlation-based distance and hierarchical clustering. In our experience, limited optimization is required here because summarizing the stratification over all imputations separately strengthens what is observed in each imputed dataset. Extra care will be required only in cases where imputations may differ significantly. After this modification, the method follows as previously described (Fig. S1).

### Data source

The data utilized in this study were obtained from two independent multicentre studies: the European PREDICT cohort and the Latin-American ACLARA cohort, conducted as part of the European Project DECISION [28, 41]. Both cohorts collected various measures, including clinical, pharmacological, biomarker, and outcome data from patients with acute decompensation of cirrhosis upon hospital admission and during follow-up visits. The follow-up period was 90 days for the PREDICT cohort and 28 days for the ACLARA cohort. To be eligible for the present study, patients were required to have acute decompensation of cirrhosis upon hospital admission, with available information on short-term outcomes, drug intake, and available biological samples. Ultimately, 766 patients from the PREDICT cohort and 580 patients from the ACLARA cohort and 74 features (continuous and categorical) were included in the analysis. The features included demographic information, clinical and laboratory data, medical history, risk factors, and cirrhosis scores at hospital admission, with missing values accounting for less than 30% (Table 1). To avoid bias from missing data, imputation was performed with 1,000 iterations using the MICE method [40].

**Table 1:** Complete list of input features. Patient characteristics included in the analysis: demographics, cause of cirrhosis, main reason for hospitalization, manifestations at admission, cirrhosis severity scores, medical history, lifestyle and laboratory variables

Demographics	Age, sex, height^+^, weight^+^, BMI, ethnicity (Black or African American, Asian, White, other)
**Cause of cirrhosis**	Alcohol, viral, alcohol + viral, NASH, cryptogenic, other
**Main reason for hospitalization**	Ascites, hepatic encephalopathy, gastrointestinal bleeding, spontaneous bacterial peritonitis, other infection
**Manifestations at admission**	Clinical Events (ascites, hepatic encephalopathy, gastrointestinal bleeding, acute kidney injury, bacterial infection, acute alcoholic-steatohepatitis, acute viral hepatitis, hepatocellular carcinoma), number of clinical events (the sum of clinical events), number of precipitating events (the sum of precipitating events: proven bacterial infection, acute alcoholic-steatohepatitis, CLIF-C AD > 50), organ dysfunction (liver, renal, cerebral, coagulation, cardiac, respiratory), number of organ dysfunctions (the sum of organ dysfunctions), organ failure (liver, cerebral, coagulation, cardiac, respiratory), number of organ failures (the sum of organ failures)
**Cirrhosis Severity Scores**	Child-Pugh, CLIF-C AD, CLIF-C OF, MELD, MELDNA
**Medical history**	History of diabetes, history of hypertension, history of previous decompensations
**Lifestyle**	Alcohol, active alcohol consumption^+^, tobacco
**Laboratory variables (measured in serum)**	Alanine transaminase, aspartate aminotransferase, albumin, bilirubin (total), gamma-GT, C-reactive protein, sodium, potassium, glucose^+^, hemoglobin, hematocrit, creatinine, white blood cell count, lymphocytes, monocytes, neutrophils, INR (International Normalized Ratio), platelet, SpO2 (%), SpO2/FiO2 Ratio

^+^Variables not included in ACLARA cohort

### ClustALL comparison to different clustering methodologies

A comparison was conducted between the ClustALL framework and classical clustering algorithms. Stratification was performed on 1,000 imputed datasets using classical k-means and hierarchical methodologies with k values of 2 and 3, considering that ClustALL robust stratifications comprised two or three patient subgroups. Bootstrapping was performed for the classical clusters to evaluate cluster-wise stability [42]. The resulting stability was compared to ClustALL stability through the Kolmogorov-Smirnov test. Moreover, the clinical utility of the various stratifications was assessed by examining the clinical insights obtained from the different clusters.

### Statistical methods

All analyses were performed in the R Computing Environment version 4.0.3 [43].

### Descriptive statistics

Descriptive characteristics of the PREDICT and ACLARA study populations were reported as means with standard deviations for continuous variables and proportions of patients for categorical variables.

### Feature analysis

The identification of the minimal-size predictive signatures with maximal predictive power leading to each stratification was performed using the *fbed.reg* function with default hyperparameters from the MXM R package [44, 45].

### Parametric tests

Differences between clusters in the PREDICT and the ACLARA cohorts were assessed using one-way ANOVA for continuous variables, while binary variables were tested with the chi-square test. The association between the PREDICT clusters identified with ClustALL –exclusively using data obtained at admission– with the groups of patients based on their clinical course [28], was tested with the Fisher test.

### Stratification model reproducibility

*AD-strat* model was validated in a separate cohort of patients with acute decompensation of cirrhosis from the ACLARA cohort and in the PREDICT follow-up time points. For this purpose, the k-nearest neighbours (kNN) model was trained on the PREDICT *AD-strat* cluster labels based on the signatures previously defined as most predictive in the feature analysis [46]. The K parameter was selected based on different measures that assessed the overall model performance over different K’s [47], including accuracy, the area under the curve (AUC), error rate (ER), false positives (FP), and false negatives (FN) (Table S7). After applying the kNN algorithm, the target data (ACLARA cohort and PREDICT follow-up) was labelled based on the majority votes from the kNN and imputed datasets. Deeper details on the kNN model and its performance evaluation are included in the Glossary in the Supplementary Methods.

### Survival analysis

Cumulative incidences of ACLF development and liver-related death were estimated using the cumulative incidence function of the survival R Package. Liver transplantation was considered a competing event. A p-value lower than 0.05 with Benjamini and Hochberg (BH) adjustment was considered statistically significant.

### Longitudinal analysis and model evaluation

All PREDICT patients with ≥ 1 post-baseline assessment (*n* = 688) were included in longitudinal outcomes analyses for a period of 90 days after hospital admission. Sankey diagrams were generated to show the patients’ transfers among the *AD-strat* clustering, liver transplant, ACLF development, death, and survival status. The predictive power of the stratification models at follow-up time points versus at baseline in the PREDICT cohort was evaluated using the Bayesian Information Criterion (BIC), the Akaike information criterion (AIC), the concordance, and the Likelihood ratio goodness-of-fit parameters [48, 49].

## Results

### ClustALL, a robust data-driven framework for patient stratification in complex diseases

We developed a specialized stratification framework, referred to as ClustALL, specifically designed to accurately identify all potential alternatives for stratifying a population using clinical multimodal data at hospital admission as input. The ClustALL methodology consists of three main steps illustrated in Fig. 1 and detailed in the Methods section: 1) **Data Complexity Reduction** (depicted in the Green Panel of Fig. 1) aims to simplify the original dataset by mitigating the impact of redundant information (highly correlated variables). As a result, we obtain a set of embeddings, each one derived from different groupings of clinical variables. 2) **Stratification Process** (depicted in the Purple Panel of Fig. 1), where, for each embedding, multiple stratification analyses are performed using different combinations of among the most widely used distance metrics and clustering methodologies (REF). From each combination, denoted as “*embedding + distance metric + clustering method*”, a stratification is derived. 3) **Consensus-based Stratifications** step (depicted in the Red Panel of Fig. 1) aims to identify robust stratifications that, in addition, exhibit minimal variation when combination parameters (“*embedding + distance metric + clustering method”*) are *slightly* modified. ClustALL performs a **population-based robustness** analysis for each stratification using bootstrapping. This analysis ensures that combinations associated with non-robust stratifications are excluded. The resulting stratifications are then compared using the Jaccard distance. As a result, a heatmap is generated to visually identify groups of representative stratifications (green squared lines). The selection of representative stratifications enables the preservation of those stratifications that demonstrate **parameter-based robustness**: consistency even when various parameters, like distance metrics or clustering methods, are altered. For each group of stratifications, the centroid is selected as the final stratification (green squares).

Combining **these three steps allows ClustALL to identify none, one, or multiple robust stratifications in a given population** of patients with complex diseases. Importantly, a specific implementation of ClustALL is designed to **effectively handle datasets with missing data effectively**, ensuring that incomplete information does not hinder the stratification process.

### ClustALL uncovers stratification in a cohort of patients with acutely decompensated cirrhosis: a proof-of-concept

#### Study population

The ClustALL approach was applied to a subset of individuals from the European PREDICT cohort [28], which included 766 patients with acute decompensation of cirrhosis and 74 clinical features collected at hospital admission, with less than 30% missing values. Complete information on patient characteristics and short-term outcomes, including acute-on-chronic liver failure (ACLF), liver transplant, and death, can be found in Supplemental Table 1.

### ClustALL identified five different alternatives to stratify the population

**Fig. 2:**
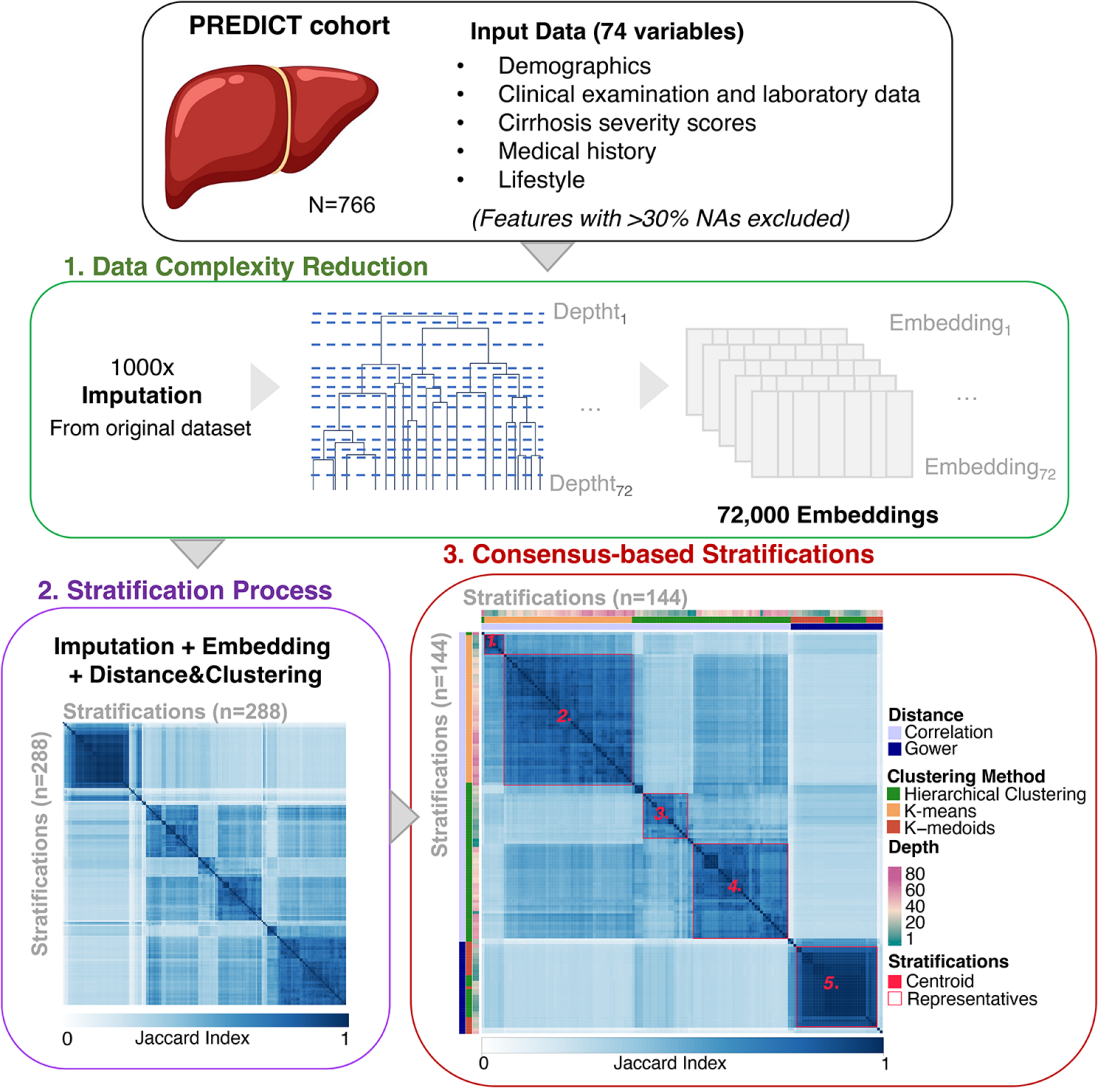
Summary of the outputs from the different steps of the ClustALL framework when applied to the PREDICT cohort (*N* = 766). Input data comprised 74 clinical features with less than 30% missing values. The analysis utilized 1,000 imputed datasets. The Data Complexity Reduction step (green) was applied to 72 depths of the 1,000 imputed datasets. The Stratification Process step (purple) considered various clustering combinations resulting in 288 stratifications. After bootstrapping, 144 robust stratifications remained. Finally, in the Consensus-based Stratification step (red), five groups of robust stratifications (red squares) were identified, and the centroid was selected from each group as the final stratifications (red coloured squares)

The ClustALL workflow was utilized to discover potential new sub-phenotypes of patients with acute decompensation of cirrhosis within the PREDICT cohort upon hospital admission (Fig. 2). To handle missing values in the dataset, we employed the ClustALL framework, which incorporates imputations using 1,000 iterations, as described in the Methods section. The Data Complexity Reduction Step resulted in 72,000 embeddings (Fig. 2). The Stratification Process generated 288 stratifications based on the different combinations of “*embedding + distance metric + clustering method*” (Fig. 2). Among these, 144 population-based robust stratifications were identified through the Consensus-based Stratifications step, resulting in five groups of parameter-based representative stratifications. The centroid was selected for each group of stratifications (Fig. 2).

### ClustALL provides better resolution than classical clustering tools

We conducted an analysis to assess the added value of ClustALL when compared with classical clustering methodologies such as k-means or hierarchical clustering. Regarding the classical methodologies, our findings revealed that when using correlation as a distance metric, 90% of patients were consistently assigned to a single cluster, regardless of the number of clusters considered; when Gower distance was utilized, the distribution of patients across clusters presented a more balanced distribution (Table S2). Notably, the population-based robustness of the stratifications generated by ClustALL was significantly higher (p-value < 0.01) compared to the results obtained using k-means and hierarchical clustering (Fig. S3). In summary, our observations demonstrate that ClustALL significantly outperforms classical methodologies regarding population-based robustness.

### Characterization of the five robust stratifications within the PREDICT population

**Fig. 3:**
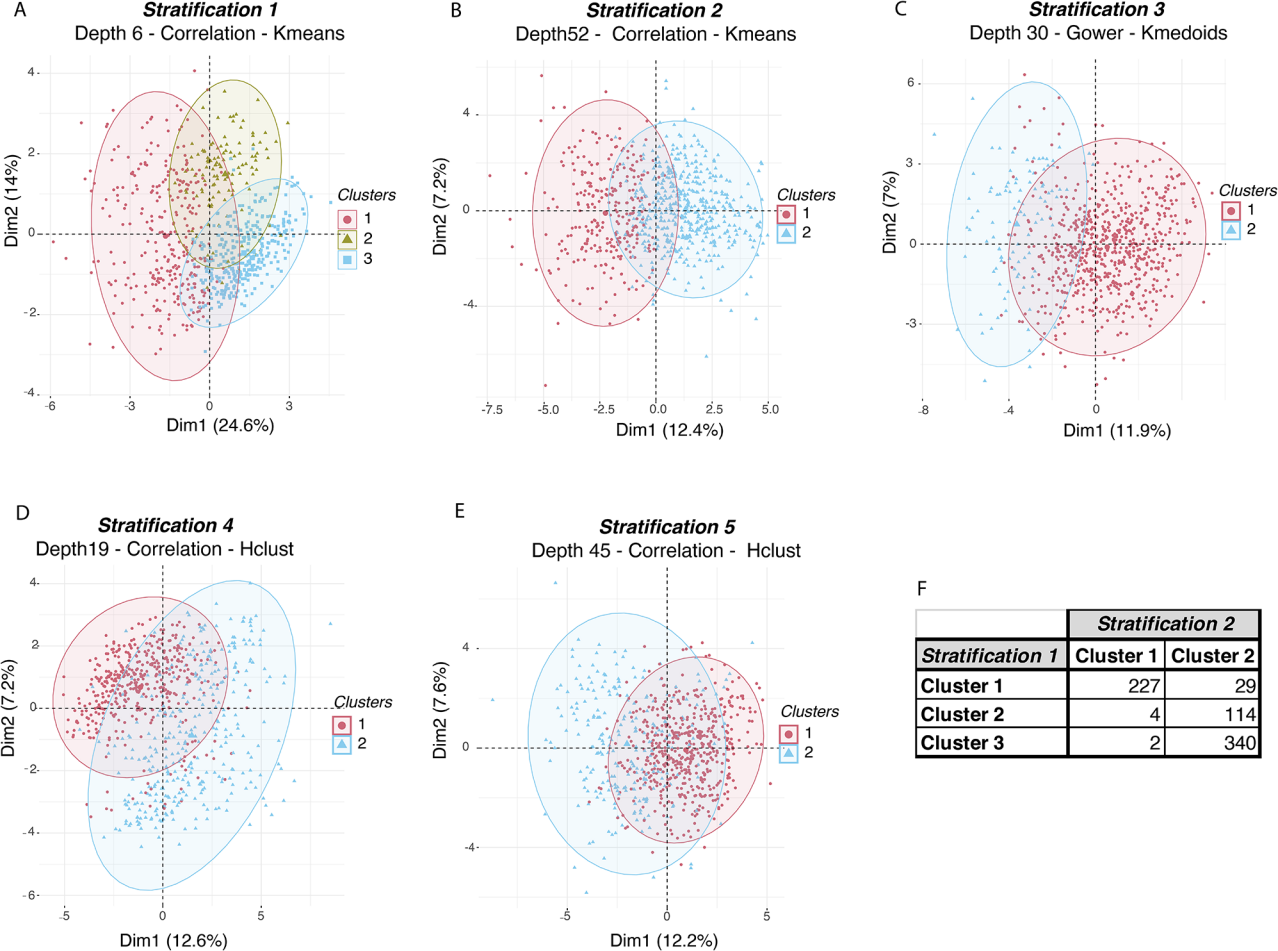
Principal Component projection of the ClustALL robust stratifications based on the embedding associated with each stratification. (**A**-**E**). Low-dimension representation of the robust stratifications after applying the ClustALL framework to the PREDICT cohort. For each one of the 5 robust stratifications identified by ClustALL, the Principal Component Analysis of the Embeddings corresponding to the specific dendrogram depth associated with the stratification is shown. The *x* (Dim1) and *y* (Dim2) axes represent the first and second principal components respectively, which are linear combinations of the original variables. (**F**). The overlap between the clusters in stratifications 1 and 2 shows that stratification 1 is a subdivision of stratification 2

**Fig. 4:**
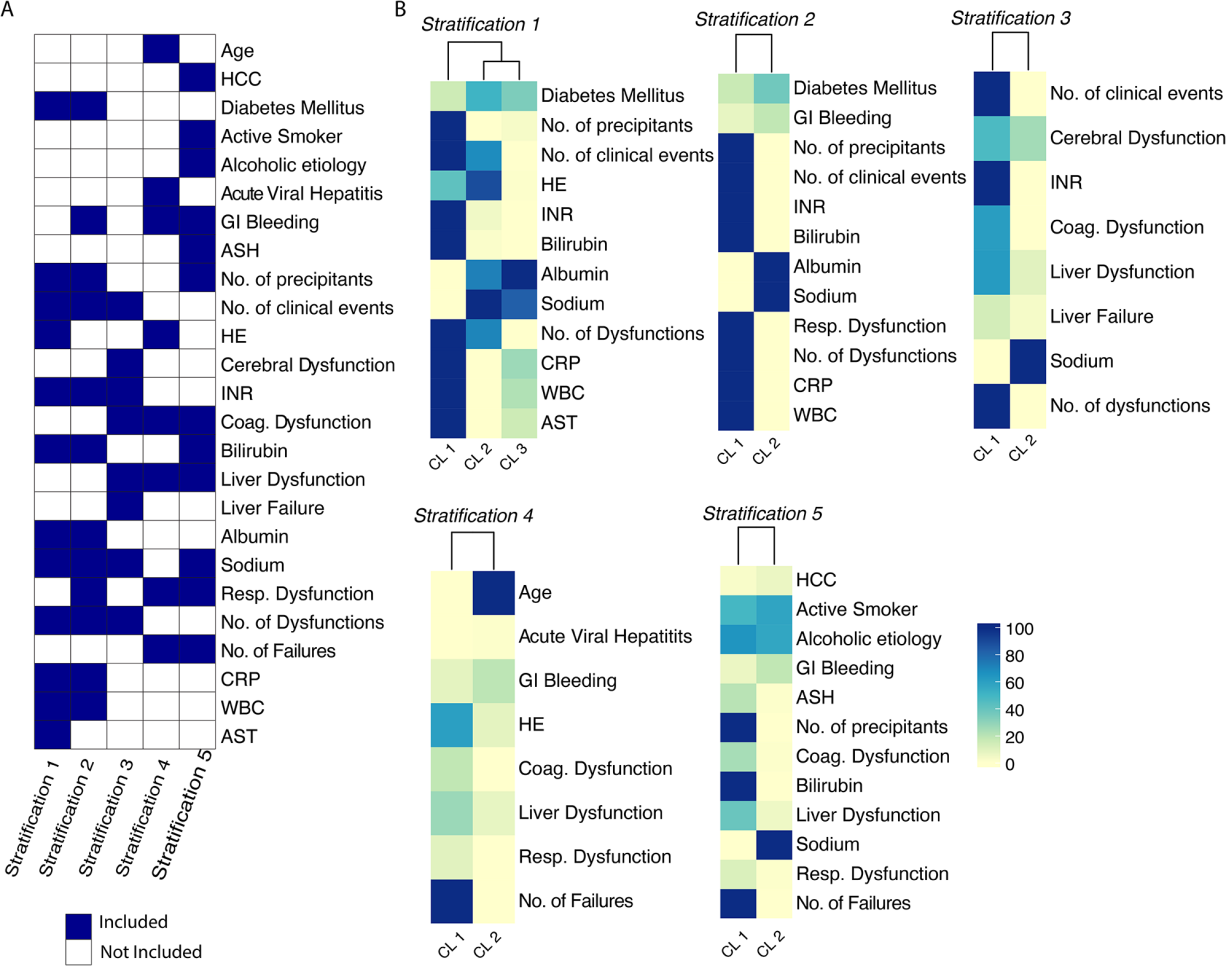
Overview of the variables driving the ClustALL stratifications. (**A**). Heatmap with the minimal set of variables required to describe the 5 different stratifications, accounting for 25 out of 74 input variables. (**B**). Heatmaps of the minimal set of patient characteristics per stratification. The heatmap colour scale depends on the data type. In the case of binary variables, the value indicates the percentage of patients with such binary characteristics, e.g., the presence of Diabetes Mellitus. For continuous variables, the colour scale represents a scaled value from the highest cluster mean (100.0) to the lowest cluster mean (0.0), e.g., Albumin and CRP. Abbreviations: ASH = Acute Alcoholic-Steatohepatitis, AST = Aspartate aminotransferase, CL = Cluster, CRP = C-Reactive Protein, HE = Hepatic encephalopathy, HCC = Hepatocellular Carcinoma, INR = International normalized ratio, WBC = White blood cell counts

After identifying the robust stratifications, we aimed to explore and characterize the distinct clusters observed in each of the five alternative stratifications. These stratifications divided the patients into two clusters, except for stratification 1, which had three clusters. We visually investigated the separation by representing each stratification in a low-dimensional space using the corresponding embeddings derived from the dendrogram depths (Fig. 3A-E) and the complete dataset (Fig. S2). Further exploration revealed that stratification 1 was a subdivision of stratification 2 (Fig. 3F). We then determined the minimal sets of variables (excluding the cirrhosis severity scores (Table S1 variables 44 to 48)) with the highest predictive performance in differentiating the clusters for each stratification (Tables S3–S7) [44, 45]. The different classification approaches were described by 25 variables from a total of 74 (Table S1 variables 1 to 74), with 8 to 12 variables per stratification (Fig. 4A). Notably, all stratifications included 3 common features: (i) serum bilirubin concentration (either as a continuous variable or categorized under the term “liver dysfunction” [50]); (ii) INR (either as a continuous variable or categorized under the term “coagulation dysfunction” [50]; (iii) the number of organ dysfunction or failure. Precipitating events were present in all but one stratification (stratification 3) either as a sum or individually (gastrointestinal bleeding, alcohol-related hepatitis, acute viral hepatitis). Diabetes mellitus was included in two stratifications. Conversely, age, sex, BMI, cause of cirrhosis, and lifestyle were present in no or one stratification. Interestingly, stratification 1 and 2 shared almost the same minimal set of variables. Both stratifications identified a group of patients with a severe phenotype attested by low serum sodium, low serum albumin, high serum bilirubin, high INR, high C-Reactive Protein (CRP) and leucocytes, and the number of precipitating events (Fig. 4B). Hepatic encephalopathy was present in stratification 1 but not in 2 [51]. A complete statistical characterization of the stratifications is provided in Tables S3 to S7. Considering the clinical implications of the features and the finer classification of the patients, we identified stratification 1 as the most insightful for further exploration in patients with acute decompensation of cirrhosis. Henceforth, in our discussions, we will refer to this specific stratification as *‘AD-strat’*.

### *AD-strat* provides prognosis value

**Fig. 5:**
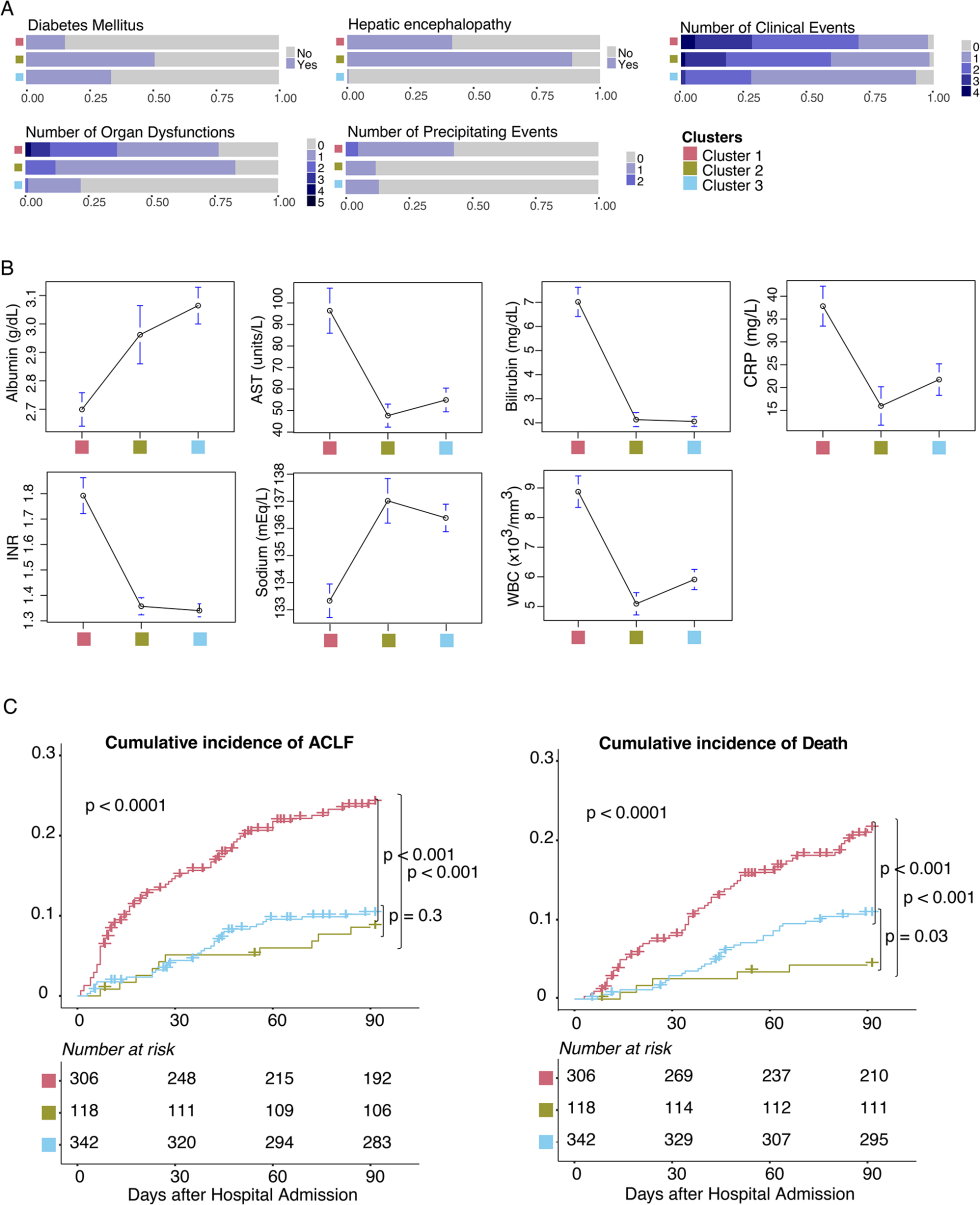
Clinical overview of the AD-strat derived clusters in the PREDICT cohort. (A, B). Distribution of the highest predictive performance-related patient characteristics among AD-strat clusters; (**A**) categorical variables, (**B**) numerical variables. (**C**) Cumulative incidence of ACLF (left) and death (right) according to the AD-strat clustering in PREDICT cohort considering 90 days after hospital admission, with the number of patients at risk per cluster (Transplantation counted as a competing risk to death). Abbreviations: AST = Aspartate aminotransferase, CRP = C- Reactive Protein, INR = International normalized ratio, WBC = White blood cell counts

The *AD-strat* stratification is defined by three subgroups (clusters) of patients with acutely decompensated cirrhosis, revealing different clinical characteristics and disease progression. Cluster 1 included 306 patients (39.95%) who exhibited the most clinically critical scenario (Fig. 5A, B and Table S3). These individuals had the highest rates of organ dysfunction, clinical events, and precipitating events (Table S3). They had a marked acute inflammatory profile (high white blood cell count and CRP level), poor liver function (low levels of albumin and high levels of INR and serum bilirubin), and more hepatocyte injury (higher levels of serum aspartate aminotransferase). Conversely, Cluster 2 (*n* = 118; 15.4%) and Cluster 3 (*n* = 342; 44.6%) had a less severe presentation. The main difference between Cluster 2 and 3 was hepatic encephalopathy, found in 89% of the patients in Cluster 2 and almost no patients in Cluster 3 (Fig. 5A, B and Table S3). Importantly, a significant prognostic value of *AD-strat* was revealed by exploring the cumulative incidence of ACLF and death over 90-day follow-up (Fig. 5C).

Liver transplantation was considered a competing event as it represents a definitive intervention that dramatically changes the course of the disease, offering a potential cure for end-stage liver disease, similarly as in other studies [28, 52, 53]. Patients in Cluster 1 had poor short-term outcomes, with a cumulative incidence of ACLF and death, both by 90 days of 24.1 and 21.5, respectively. While Clusters 2 and 3 had similar risks of ACLF by 90 days (8.6% and 10.2%, respecively), the risk of death by 90 days was lower for Cluster 2 than Cluster 3 (4.3% vs. 10.7%). When we compared the clusters identified with ClustALL - exclusively using data obtained at admission - with the groups of patients based on their clinical course [28], we found a statistically significant association (Fisher test, p-value < 0.01) (see Table S8). We observed that 61% of patients with pre-ACLF were in Cluster 1, and 48% of patients with stable decompensated were in Cluster 3.

### Reproducibility of the stratification model in an independent cohort

**Fig. 6:**
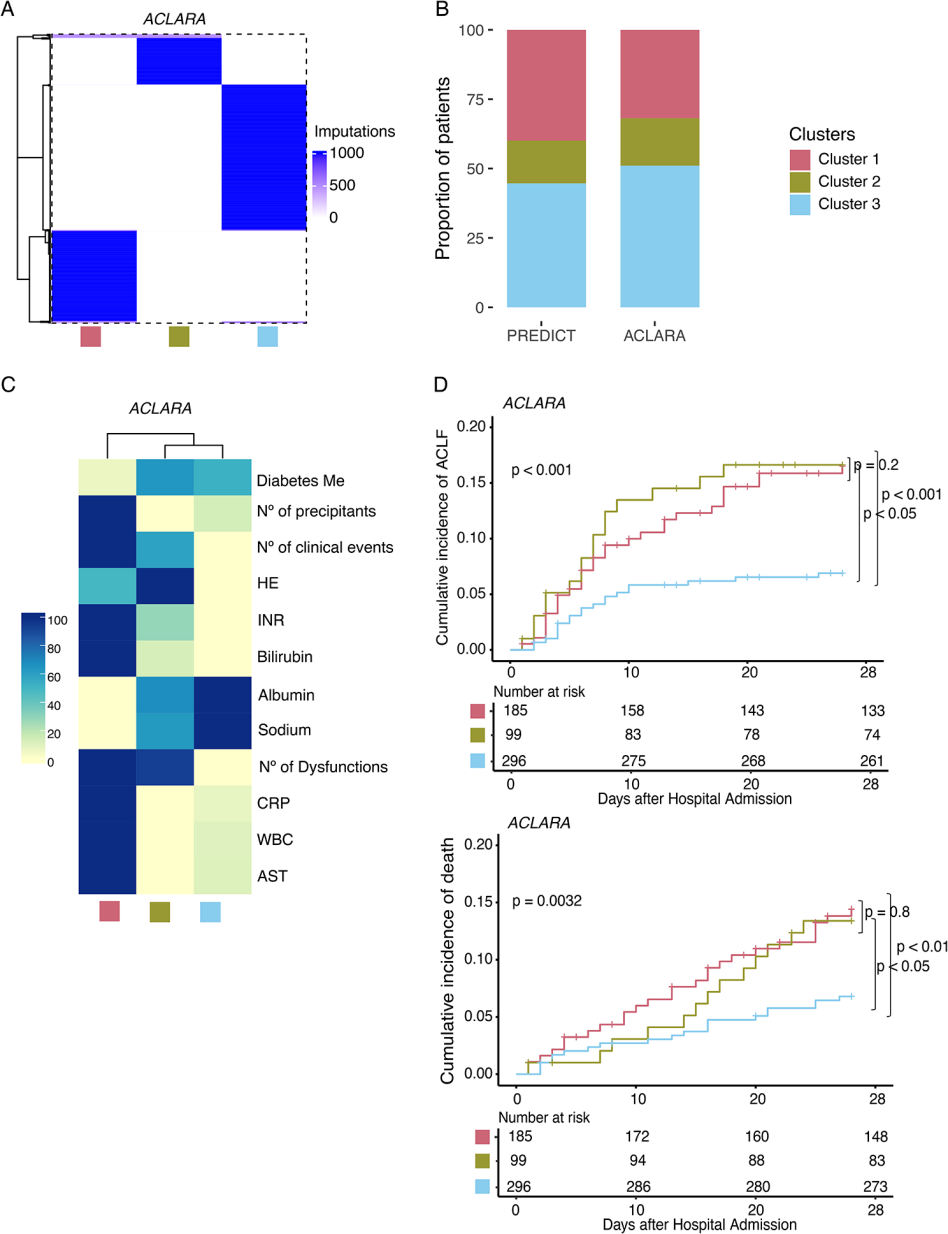
Reproducibility of the AD-strat model in the ACLARA cohort. (**A**) Distribution of the labels in the ACLARA cohort after applying the kNN model 1,000 times. (**B**) Proportion of patients distributed in the 3 clusters in the PREDICT and the ACLARA cohorts. (**C**) Heatmap of patient characteristics per cluster in the ACLARA cohort. Bars on the right show the colour scale representing the proportion with each binary characteristic, such as diabetes. Continuous variables, such as bilirubin, represent a scaled value from the highest cluster mean (1.0) to the lowest cluster mean (0.0). (**D**) Cumulative incidence of ACLF (up) and death (down) according to the AD-strat clustering in ACLARA cohort considering 28 days after hospital admission, with the number of patients at risk per cluster (Transplantation counted as a competing risk to death). Abbreviations: AST = Aspartate aminotransferase, CRP = C- Reactive Protein, INR = International normalized ratio, WBC = White blood cell counts

We assessed the validity of the *AD-strat* model in a large independent prospective multicentre cohort that included 580 patients with acute decompensation of cirrhosis from the Latin-American ACLARA study [41]. Using as a reference the PREDICT *AD-strat* clusters, we labelled ACLARA patients using the k-nearest neighbours classification algorithm (Table S10) [46]. The classification model included the 12 predictive variables previously identified in the feature importance analysis (Fig. 4B Stratification 1). Importantly, the allocation of the patients to the clusters was consistent and independent of the imputation in 99% of the cases (Fig. 6A), and the distribution of individuals by *AD-strat* clusters within ACLARA closely mirrored that of the PREDICT cohort (Fig. 6B). As expected, the clustering of the ACLARA cohort exhibited similar clinical feature patterns to the PREDICT cohort (Figs. 4B and 6C Stratification 1). Furthermore, the features describing the subgroups demonstrated statistical significance (Table S11). Finally, we assessed the clinical relevance of the clustering in terms of prognosis, specifically examining the short-term outcomes available in the ACLARA cohort 28 days after hospital admission. Similar to results obtained in the PREDICT cohort, Cluster 1 displayed a bad prognosis for both ACLF and death, while Cluster 3 showed a better prognosis (Fig. 6D). In ACLARA, all patients from Cluster 2 were afflicted by hepatic encephalopathy (Table S11) and showed a poor prognosis similar to that of Cluster 1. Ethnicity was homogeneously distributed across clusters (Table S2). In particular, Native Americans represented 21% of Cluster 1, 15% of Cluster 2, and 14% of Cluster 3. Complete information on patient characteristics and short-term outcomes is reported in Supplemental Table 9.

### *AD-strat* as a marker for clinical management

**Fig. 7:**
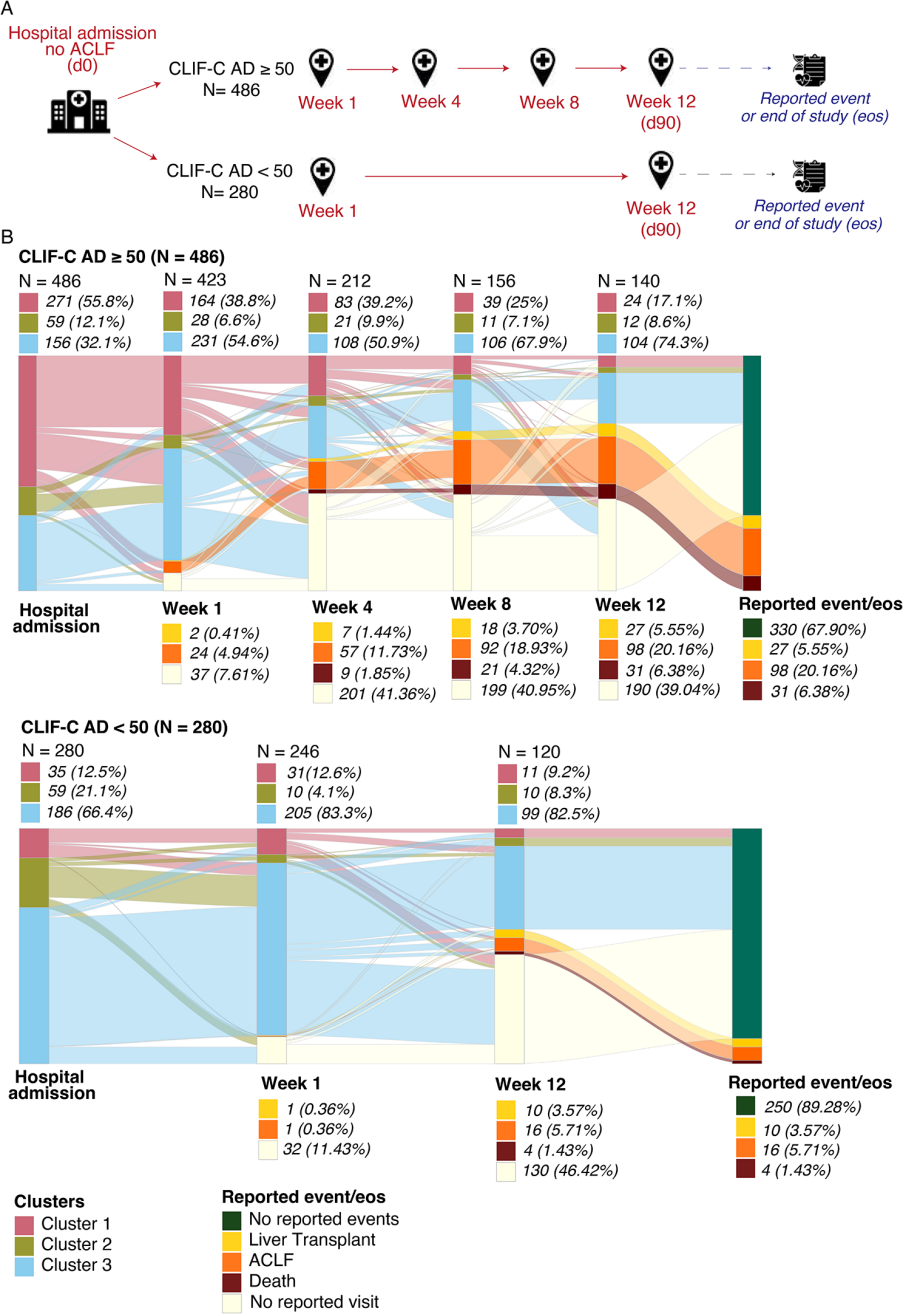
Distribution and transition of the AD-strat derived clusters at different visits in the PREDICT cohort. (**A**). Schematic representation of PREDICT study design. Two follow-up visit plans were defined according to the reported disease severity (CLIF-C AD-score) at hospital admission (red). The information about the occurrence of any adverse event (liver transplant, ACLF or death) during the whole visit plan or the absence of events at the end of the study was tracked (blue). (**B**) Sankey plots show the cluster label of each patient over the follow-up visits. The follow-up flows of patients with CLIF-C AD > = 50 at hospital admission (up) and CLIF-C AD < 50 at hospital admission (down) are shown. The distribution of the patients assessed at each follow-up visit per cluster is shown as frequency and proportion on the top of the Sankey representations. The accumulated frequency and proportion of adverse events at each follow-up visit respecting the whole cohort (for CLIF-C AD > = 50, *n* = 486; for CLIF-C AD < 50, *n* = 280) are shown on the bottom of the Sankey representations. Reported event/end of study (EOS), shows the status of a patient at the “end of the study”: patients with a reported event or patients with no reported event

Finally, we investigated the clinical value of the stratification during the follow-up visits of the PREDICT cohort. Based on the PREDICT study design [28], two follow-up visit plans were established depending on the reported disease severity (CLIF-C AD-score) at hospital admission (Fig. 7A). For patients with a CLIF-C AD-score ≥ 50, the scheduled visits were performed at hospital admission and 1, 4, 8 and, 12 weeks after enrolment. For patients with a CLIF-C AD-score < 50, the scheduled visits were performed only at hospital admission and 1 and 12 weeks after enrolment.

Of the 766 patients included in the PREDICT study, 688 had at least one follow-up visit. For this subset of patients with available data, we labelled each of them at each follow-up visit using the kNN algorithm (Fig. 7B). This approach allowed an overview of the patient stratification over the entire study duration and revealed the patient flow over time, highlighting cluster transitions.

Consistent with the previous *AD-strat* characterization at hospital admission (Fig. 5 and Table S3), we identified more than 50% of patients with a CLIF-C AD score ≥ 50 (*n* = 486) were classified as Cluster 1, while patients with CLIF-C AD score < 50 (*n* = 280) were predominantly classified as Cluster 3 (66.4%) (Fig. 7B). Changes in cluster proportions were observed during the patients’ follow-up. Stratification changes over time were more pronounced among patients with a CLIF-C AD scores ≥ 50 at hospital admission, showing a progressive reduction of patients classified as Cluster 1 (55.8% at hospital admission, 38.8% at week 1, 39.2% at week 4, 25% at week 8, and 17.1% at week 12) and an increase of those classified as Cluster 3 (32.1% at hospital admission, 54.6% at week 1, 50.9% at week 4, 67.9% at week 8, and 74.3% at week 12). Additionally, there was a progressive increase in the proportion of patients classified as Cluster 3 for those patients with a CLIF-C AD-score < 50 at hospital inclusion (66.4% at hospital admission, 83.3% at week 1, and 82.5% at week 12).

To assess the effectiveness of the *AD-strat* throughout disease progression, we determined its prognostic value in two scenarios: (1) using the stratification at hospital admission, and (2) using the stratification at the last visit reported before the occurrence of any adverse event (we considered any visit between week 1 and 12) or at the end-of-study (EOS) (week 12 visit). A significant difference was observed (*p* < 0.001, Wilcoxon test) when comparing the time window between the visit used in each scenario and the occurrence of adverse events (Fig. S5), indicating that in the second scenario, we evaluated patients during a visit much closer to the event.

**Fig. 8:**
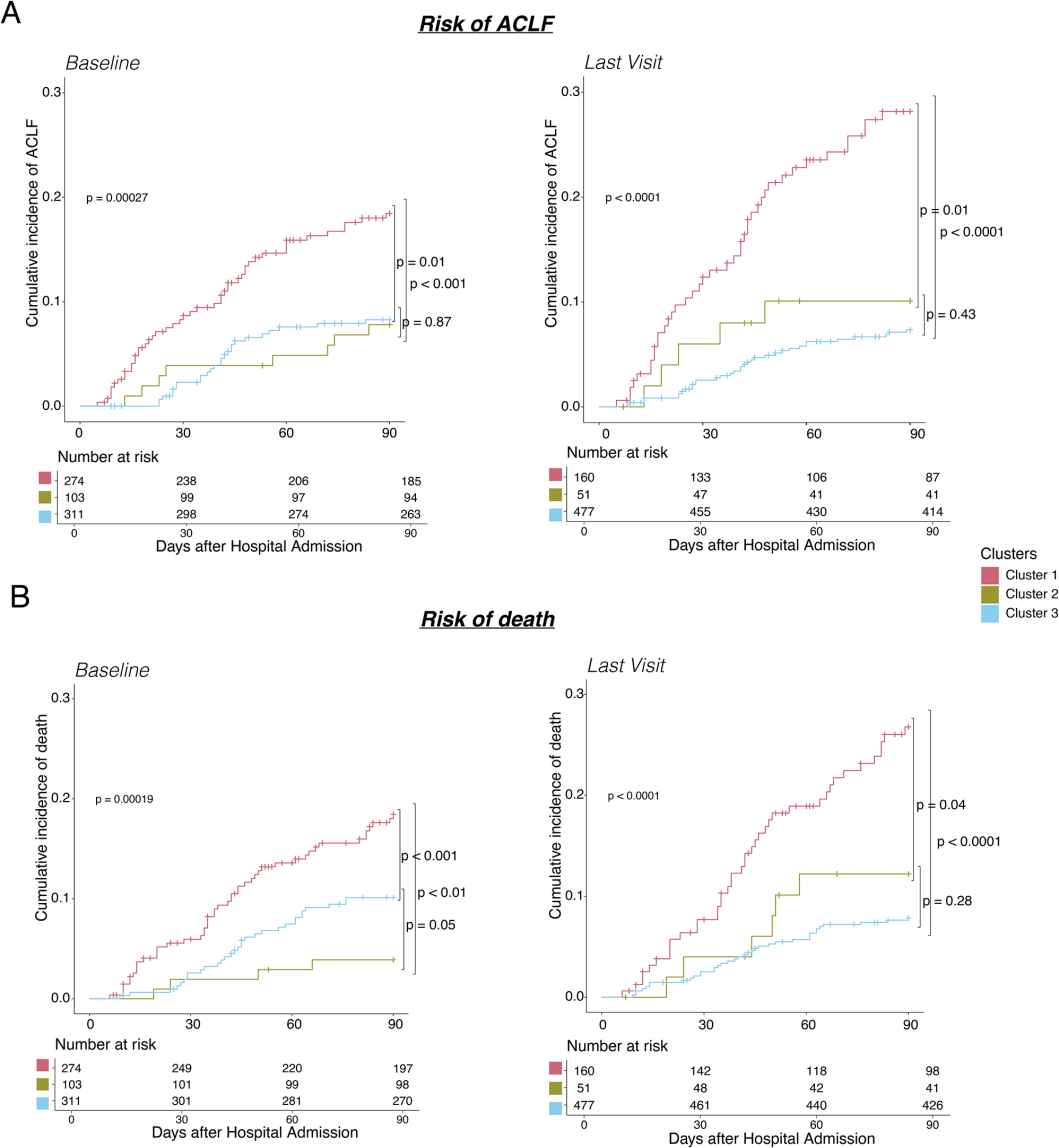
Assessment of the risk of adverse events according to the AD-strat clusters at different time points. (**A**, **B**). Cumulative incidence of ACLF (**A**) and death (**B**) according to the AD-strat clustering in PREDICT cohort at hospital admission (left) versus at last visit (right) considering 90 days after hospital admission, with the number of patients at risk per cluster (Transplantation counted as a competing risk to death)

Ultimately, the cumulative incidence of ACLF and death as stratified at the last visit demonstrated a more significant separation between clusters compared to patient stratification at hospital admission (Fig. 8). There was an increase in the incidence for those patients classified as Cluster 1 (18.46% and 18.45% at baseline and 28.16% and 26.8% at the last visit for ACLF and death, respectively). Accordingly, the goodness-of-fit parameters indicated an improvement in risk prediction with the last visit stratification, suggesting an enhanced predictive power as the event approached (Table S12).

## Discussion

In the current era of personalized medicine, there is a growing focus on elucidating the complexities of disease populations, reflecting an emphasis on understanding their inherent heterogeneity [54–56]. Consequently, both academic and clinical efforts have been dedicated to characterizing disease subtypes for the purposes of identification, treatment, and prognosis. Or more general, aiming to enhance our understanding and management of complex conditions. Furthermore, the WHO has recognized patient stratification as an invaluable approach [8]. It is important to note that patient stratification extends beyond mere outcome prediction scores, particularly in scenarios where a “one-size-fits-all” approach to treatment may inadequately address the diverse needs and characteristics of individual patients [57].

Patient stratification, as investigated in this study, involves the unsupervised grouping of patients based on available clinical data. Interestingly, while significant progress has been made in classification problems, particularly in domains like single-cell transcriptomic analysis [58, 59], unsupervised clustering of patients based on clinical information is still in the developmental stage [9, 60]. Notably, the existing challenges in clinical stratification, such as handling mixed data types, missing values, or highly correlated variables, are often mitigated using ad-hoc solutions, given the absence of a comprehensive method to address them. To overcome the aforementioned idiosyncrasies, we have developed a novel computational framework named ClustALL.

During the development of ClustALL, our focus extended beyond simply generating patient groups; we were equally invested in ensuring the robustness of the identified stratifications. Typically, clustering robustness can be evaluated based on the stability of the clusters when modifications are made to the population using methods such as resampling or bootstrapping (population-based robustness). Significantly, ClustALL incorporates a second, less explored but equally important, dimension of robustness: assessing the consistency among the resulting stratifications when minor modifications are applied to the clustering parameter settings. This property has already been explored in the context of gene expression data as the “propensity of a clustering algorithm to maintain output coherence over a range of settings” [61]. Another major feature of ClustALL, is its capacity to identify more than one robust stratification within a given population. Clinical data is complex and allows for multiple uses and “multiple interpretations” that *may* result in several *valid* groupings [62]. Indeed, the concept of “multiple interpretations” arises from how variables are utilized in the clustering process and has been a research subject in the last decades [63]. Traditional methods such as k-means or hierarchical clustering typically yield a single outcome, which may be influenced by random initial conditions at the start of the algorithm. We consider that any stratification method should allow for the identification of multiple solutions, necessitating clinical feedback to ascertain their relevance. In contrast to traditional methods, following trace-based clustering principle, ClustALL does not rely on a random single initialization of the clustering, but, in general, integrates the information of multiple clustering efforts and evaluation criteria [18]. In summary and considering all these factors, we believe that ClustALL represents a necessary step towards practical unsupervised patient stratification; notably through the incorporation of parameter-based robustness and its capacity to identify more than one stratification.

To assess the effectiveness of ClustALL, we applied it as a proof-of-concept in a cohort of patients with acutely decompensated cirrhosis, considering clinical data collected at hospital admission. Such an attempt to apply a data-driven stratification to patients with cirrhosis has never been conducted. The stratification we set up differs from the scores developed and routinely used in patients with cirrhosis (e.g., MELD, MELD-Na, Child-Pugh, CLIF-C-AD) both in terms of design and use. Indeed, all these scores were built using a follow-up endpoint (usually death) in patients receiving therapies. These scores are helpful in identifying patients at high risk of poor outcomes. Still, they do not fully capture the heterogeneity of the patients at admission for several reasons: (a) some features explaining patients’ heterogeneity might not have an independent prognostic value, either because the prognostic information they carry is contained in other variables, or because therapies administered to patients during their follow-up blunt their impact; (b) a similar survival rate does not imply similar pathophysiological mechanisms. For instance, in PREDICT, clusters 2 and 3 have a similar rate of ACLF, while they strongly differ with regard to the prevalence of hepatic encephalopathy.

In the first step of our analysis, we identified five alternative stratifications for patients with acute decompensation of cirrhosis. Interestingly, all stratifications included markers of impaired liver function, serum bilirubin and INR, and the number of organ dysfunction or failure, and all but one included precipitating events. This emphasizes that these features are crucial when designing a clinical trial, including patients with acute decompensation of cirrhosis. Our data-driven approaches show that serum bilirubin and INR are not only key to predicting the outcome of patients with cirrhosis but also to explaining heterogeneity at admission. On the contrary, features like age, sex, BMI, cause of cirrhosis, and lifestyle were present in no or only one stratification, suggesting that these features are not key when designing a clinical trial. The stratification we selected (*AD-strat*) provided a more granular resolution by allowing the identification of three subgroups of patients.

In this stratification, diabetes mellitus is taken into account. While it is known that diabetes is an independent risk factor for cirrhosis decompensation [64, 65], the role of diabetes once acute decompensation has happened has been overlooked so far. This place of diabetes is quite unique since causes of cirrhosis, comorbidities, or lifestyle were not part of the key features of *AD-strat*. Hepatic encephalopathy strongly impacted the categorization of patients with acutely decompensated cirrhosis. Notably, 89% and 100% of the patients in Cluster 2 from the PREDICT and ACLARA cohorts, respectively, presented hepatic encephalopathy at the time of hospital admission. This may explain the intermediate prognosis observed in patients within Cluster 2, as hepatic encephalopathy is recognised by its fluctuating nature and potential reversibility [66, 67]. The dynamic nature of hepatic encephalopathy may also explain why Cluster 2 was not a static group over time [68].

The stratification presented here is not intended to guide clinical bedside decisions or to create a new prognostic score, but rather to identify *more* homogeneous patient populations upon hospital admission. However, once we applied ClustALL, we observed that the three subgroups of patients identified had a different outcome. Moreover, employing *AD-strat* labelling over time facilitated dynamic and enhanced identification of high-risk patients in the PREDICT cohort. These findings underscore ClustALL’s ability not only to stratify patients based on baseline characteristics, with a prognostic relevance. In this regard, *AD-strat* might be a useful tool for designing future clinical trials by including more homogeneous patient populations. Using ClustALL may also offer insights into applying nanomedicine in precision-targeted drug delivery systems [69, 70]. Furthermore, we have implemented an online calculator for acutely decompensated cirrhosis based on this stratification output, available at https://decision-for-liver.eu/for-scientists/clustall-web-application/.

Although our study showed promising results, it is important to acknowledge some limitations. Firstly, concerning our novel stratification framework, we designed a method aimed at minimizing user-defined parameters by exhaustively exploring all potential clustering solutions across various parameter combinations. However, practical decisions were made, such as employing PCA to diminish the dimensionality of highly correlated variables. In future iterations, we intend to explore alternatives such as Independent Component Analysis or PCA tailored for ordinal variables. Additionally, the determination of the number of components included in each dimensionality reduction will be guided by data-driven criteria. Furthermore, the ClustALL framework offers scope for expansion by incorporating additional methods and distance metrics, affording users the autonomy to select those most suitable for their needs. Secondly, our stratification relied solely on routinely available clinical data collected at hospital admission, potentially limiting the comprehensive understanding of patients’ conditions. Future investigations should integrate biological data, preferably derived from multiomic analyses. It is also relevant to note that in the ACLARA cohort, predictive power was assessed only at the 28-day mark due to study design constraints. Moreover, it is worth highlighting that broader utilization of ClustALL (e.g., in other complex diseases and/or including omic data) may shed light on areas necessitating refinement, aligning with the *No-Free Lunch* theorem discussed previously [21].

In summary, this study introduces a novel unsupervised clustering framework, ClustALL, capable of overcoming the limitations of available stratification methods. Expanding beyond cirrhosis, ClustALL shows potential for wider implementation across various clinical settings, hinting at its ability to transform patient subgroup identification, expand possibilities of drug repurposing [71], and in general, to enhance healthcare management.

## Conclusions

ClustALL stands out as a comprehensive and versatile computational framework for unsupervised patient stratification that uses multimodal clinical data such as biochemical markers, demographics, and clinical scores as input. Furthermore, ClustALL ensures the identification of robust stratifications—including two concepts of robustness—and allows the identification of multiple robust stratifications over the same population. In the context of acute decompensation of cirrhosis, ClustALL not only successfully navigates the intricacies of diverse clinical information but also identifies several robust stratifications. Furthermore, validating findings across different time points and in an independent cohort underscores the reliability of ClustALL. Overall, this work not only contributes to our understanding of patient heterogeneity in cirrhosis but also positions ClustALL as a powerful stratification tool that could be applied to other diseases, thereby advancing precision medicine and facilitating the development of more targeted and effective clinical interventions. Future developments of the tool could expand ClustALL framework by incorporating biological data from multiomic analyses and offering further customizable user functions.

### Electronic supplementary material

Below is the link to the electronic supplementary material.

Supplementary Material 1

Supplementary Material 2

Supplementary Material 3

Supplementary Material 4

Supplementary Material 5

Supplementary Material 6

## Data Availability

Researchers who provide a methodology sound proposal can apply for the data, as far as the proposal is in line with the research consented by the patients. These proposals should be requested through https://www.clifresearch.com/decision/Home.aspx. Data requestors will need to sign a data transfer agreement. The code to generate the ClustALL method is available on GitHub, at https://github.com/TranslationalBioinformaticsUnit/ClustALL_AD/.

## Abbreviations

ACLF: Acute-on-chronic liver failure
AD: Acute decompensation of cirrhosis
AIC: Akaike information criterion
AUC: Area under the curve
BIC: Bayesian Information Criterion (BIC)
BH: Benjamini and Hochberg
CRP: C-reactive protein
EOS: End of study
ER: Error rate
FN: False negatives
FP: False positives
INR: International Normalized Ratio
kNN: k-nearest neighbors
MICE: Multivariate imputation by chained equations
PC: Principal component
PCA: Principal component analysis
