# Supplementary material for: A robust clustering strategy for stratification unveils unique patient subgroups in acutely decompensated cirrhosis
Source: J Transl Med. 2024 Jun 27;22:599. doi: 10.1186/s12967-024-05386-2 (PMC11210156; doi:10.1186/s12967-024-05386-2)
Supplement: Supplementary file 1 — Supplementary Material 1 [file 12967_2024_5386_MOESM1_ESM.docx]

**A robust clustering strategy for stratification of patients with acute decompensation of cirrhosis**

**Supplementary Material**

**Glossary of Technical Terms**

This section is dedicated to enhancing the understanding of key technical concepts crucial for the development and utilization of the ClustALL framework. Each definition provided here is tailored to align with the methodology and principles underlying the ClustALL, ensuring clarity and relevance to our approach.

***Bootstrapping***

Bootstrap resampling involves randomly sampling subsets of the original dataset with replacement to create multiple samples. Each bootstrap sample is then clustered independently using the same clustering algorithm. The resulting cluster assignments are then aggregated or combined across all bootstrap samples to assess the stability of the clusters, or in other words, to assess how many times two items fall in the same cluster. This resampling technique is particularly useful when the sample size is limited, or the underlying population distribution is unknown (1–3)

***Cluster-wise stability***

The main objective of clustering is to create groups of items that are highly similar to each other and dissimilar to other groups (4). Stability measures attempt to capture how well partitions and clusters are preserved when the original dataset is perturbed. Cluster stability refers to the consistency of cluster assignments when the data is perturbed or when different subsets of the data are used. Bootstrapping can be used to assess cluster stability by repeatedly resampling the data and evaluating the consistency of cluster assignments across different bootstrap samples (5,6).

***Dendrogram and Depth***

A dendrogram is a diagram that shows hierarchical relationships between items. Its interpretation revolves around the concept of "depth". The depth indicates the distance at which two items are joined together in the dendrogram. This distance reflects the degree of similarity or dissimilarity between the items, with similar or correlated variables tending to cluster together. The closer the depth level is to the root of the dendrogram, the more similar or correlated the items are. Cutting the dendrogram at different depth levels allows for the creation of groups of variables of varying sizes (7–10).

***Embedding***

Embedding refers to mapping high-dimensional data into a lower-dimensional space, which is more compact while preserving the structural relationships or properties of the original data. It facilitates and makes subsequent analysis more efficient (10).

In the context of ClustALL, a dendrogram illustrates the relationship among the input variables, clustering them based on their similarity. Rather than selecting a particular variable from a cluster of similar ones, we consolidate them using an embedding. As mentioned earlier, by cutting the dendrogram at different depth levels, we can form groups of correlated variables of different sizes. Our method entails evaluating all possible depths of the dendrogram and summarizing clusters of items at each depth by computing embeddings.

One significant advantage of this strategy is that it avoids the need to select specific variables among the correlated ones. Traditional variable selection methods may lead to information loss, especially when dealing with clinical data and highly correlated variables. By summarizing the variables using embeddings, we can preserve more information about the original data while still capturing the underlying structure revealed by the dendrogram.

***Jaccard Index***

The Jaccard index, a metric frequently employed in data analysis, quantifies the similarity between two sets by evaluating the ratio of their shared elements to the total number of unique elements across both sets. Specifically, it compares the intersection (the common elements) to the union (the combined unique elements) of the two sets. In the scenario of evaluating clusters derived from bootstrap samples, the Jaccard index serves as a valuable tool for measuring the similarity between pairs of clusters obtained from different iterations of the bootstrap procedure. This facilitates the assessment of cluster stability and provides insights into the consistency of cluster assignments across multiple resampled datasets (11,12).

***K-Nearest Neighbour (kNN)***

*kNN* is a non-parametric algorithm that uses proximity to classify an individual data point. When used for classification problems, kNN predicts the class membership of a data point by considering the classes of its nearest neighbors. The majority class among the k nearest neighbors is assigned to the data point. When the task consists of a regression problem, kNN predicts the value of a continuous target variable by averaging the values of its nearest neighbors. The average (or weighted average) of the target variable among the k nearest neighbors is assigned as the predicted value. It is important to mention that the KNN algorithm belongs to the category of "lazy learning" models, which implies that it solely retains a training dataset without undergoing a dedicated training phase. Consequently, all computation takes place at the time of classification or prediction. Due to its reliance on memory for storing training data, it is also known as an instance-based or memory-based learning approach (13,14).

Evaluating supervised learning models such as *kNN* is an important step in ensuring their accuracy and generalizability (15). There exist several metrics available for assessing supervised learning models, with some of the frequently utilized ones being:

***Confusion matrix and classification metrics***

A *confusion matrix* is a tabular representation that compares the number of correct and incorrect predictions made by a model (like kNN) with the actual labellings in the data (15). The performance of such models is commonly evaluated using metrics extracted from the confusion matrix.

- *True Positives (TP):* Instances that have been correctly predicted as positive by the model.
- *True Negatives (TN)*: Instances that have been correctly predicted as negative by our model.
- *FP (False Positives):* Actual Negatives in data, but the model has predicted them as positive (Type I error).
- *FN (False Negatives):* Actual Positives in data, but the model has predicted them as negative (Type II error).

*Accuracy* is a metric derived from the confusion matrix. It represents the percentage of correctly predicted values from all the data points. While accuracy is a useful metric, it may not be sufficient to assess a model's performance in scenarios where the classes are imbalanced or when different types of errors have different consequences.

*Area under de curve* (AUC) is a metric commonly used to evaluate the performance of binary classification models, particularly in the context of receiver operating characteristic (ROC) curves. The ROC curve is a line graph reporting the true positive rate (sensitivity) on the y-axis and the false positive rate (1 - specificity) on the x-axis, for different threshold values. The AUC quantifies the overall performance of the classifier by calculating the area under the resulting ROC curve, ranging from 0 to 1. An AUC of 1 signifies a perfect classifier, while an AUC of 0.5 indicates performance equivalent to random chance.

*Error rate* (ER) refers to the degree of prediction error of a model made with respect to the true labels. is typically calculated as the ratio of misclassified instances to the total number of instances in the dataset, expressed as a percentage. Lower error rates indicate better performance.

***Principal Component Analysis***

Principal Component Analysis (PCA) is a dimensionality reduction technique that transforms high-dimensional data into a lower-dimensional space while preserving the most important information. It achieves this by identifying the principal components, which are orthogonal vectors that capture the maximum variance in the data (16–19).

Taking the first three principal components in PCA is often done because they collectively capture a significant portion of the variance present in the data. In some applications, the first three principal components may correspond to the most meaningful or interpretable patterns in the data, making them particularly useful for downstream analysis or modelling.

In ClustALL, PCA is applied to the groups of variables obtained from the dendrogram to further enhance the clustering process. Specifically, after identifying clusters of variables based on their relationships within the dendrogram, PCA is utilized to reduce the dimensionality of each cluster. This reduction facilitates the extraction of key features or patterns.

***Survival model evaluation***

In survival analysis, model evaluation is crucial for identifying the model that best captures the relationship between covariates and survival times while avoiding overfitting. Parameters like Bayesian Information Criterion (BIC), Akaike Information Criterion (AIC), concordance, or Likelihood ratio provide quantitative measures of model fit and complexity, helping to identify the model that strikes the best balance between goodness of fit and parsimony (20,21).

*BIC* balances the model's goodness of fit with its complexity, penalizing complex models to avoid overfitting. The lower the BIC value, the better the model is considered.

*AIC* is another model selection criterion similar to BIC but with a smaller penalty for complex models. Like BIC, it balances model fit and complexity, aiming to select the model that minimizes information loss. A lower AIC value indicates a better model fit.

*The concordance index* measures the proportion of pairs of individuals where the individual with the higher predicted risk also has the shorter survival time. A concordance value of 0.5 indicates random chance, while a value closer to 1 indicates better predictive accuracy.

*The Likelihood Ratio* assesses whether adding additional parameters to a model significantly improves its fit. The likelihood ratio test statistic is calculated as the difference in the log-likelihoods of the two models, scaled by the standard error of the difference. If the test statistic exceeds a critical value, it suggests that the more complex model provides a significantly better fit to the data than the simpler model.

1. Jain AK, Moreau JV. Bootstrap technique in cluster analysis. Pattern Recognit. 1987 Jan;20(5):547–68.

2. Fang Y, Wang J. Selection of the number of clusters via the bootstrap method. Comput Stat Data Anal. 2012 Mar;56(3):468–77.

3. Yu H, Chapman B, Di Florio A, Eischen E, Gotz D, Jacob M, et al. Bootstrapping estimates of stability for clusters, observations and model selection. Comput Stat. 2019 Mar 28;34(1):349–72.

4. Kaufman L, Rousseeuw PJ. Finding Groups in Data. Wiley; 1990.

5. Liu T, Yu H, Blair RH. Stability estimation for unsupervised clustering: A review. WIREs Computational Statistics. 2022 Nov 9;14(6).

6. Hennig C. Cluster-wise assessment of cluster stability. Comput Stat Data Anal. 2007 Sep;52(1):258–71.

7. Chavent M, Genuer R, Saracco J. Combining clustering of variables and feature selection using random forests. Commun Stat Simul Comput. 2021 Feb 1;50(2):426–45.

8. Wu J, Xiong H, Chen J. Towards understanding hierarchical clustering: A data distribution perspective. Neurocomputing. 2009 Jun;72(10–12):2319–30.

9. Schonlau M. Visualizing non-hierarchical and hierarchical cluster analyses with clustergrams. Vol. 19, Computational Statistics. 2004.

10. Chehreghani MH. Hierarchical Correlation Clustering and Tree Preserving Embedding. 2020 Feb 18; Available from: http://arxiv.org/abs/2002.07756

11. Fletcher S, Islam Z. Comparing sets of patterns with the Jaccard index. Vol. 22, Australasian Journal of Information Systems Fletcher & Islam. 2018.

12. Tang M, Kaymaz Y, Logeman BL, Eichhorn S, Liang ZS, Dulac C, et al. Evaluating single-cell cluster stability using the Jaccard similarity index. Bioinformatics. 2021 Aug 9;37(15):2212–4.

13. Deng Z, Zhu X, Cheng D, Zong M, Zhang S. Efficient kNN classification algorithm for big data. Neurocomputing. 2016 Jun 26;195:143–8.

14. Singh M, Kataria A, Singh MD. A Review of Data Classification Using K-Nearest Neighbour Algorithm [Internet]. Vol. 9001, Certified Journal. 2008. Available from: https://www.researchgate.net/publication/353306410

15. Ali N, Neagu D, Trundle P. Evaluation of k-nearest neighbour classifier performance for heterogeneous data sets. SN Appl Sci. 2019 Dec 6;1(12):1559.

16. Abdi H, Williams LJ. Principal component analysis. Vol. 2, Wiley Interdisciplinary Reviews: Computational Statistics. 2010. p. 433–59.

17. Coste J, Boueé S, Ecosse E, Leplè A, Pouchot J. Methodological issues in determining the dimensionality of composite health measures using principal component analysis: Case illustration and suggestions for practice.

18. Jollife IT, Cadima J. Principal component analysis: A review and recent developments. Vol. 374, Philosophical Transactions of the Royal Society A: Mathematical, Physical and Engineering Sciences. Royal Society of London; 2016.

19. Ringnér M. What is principal component analysis? [Internet]. Vol. 26, NATURE BIOTECHNOLOGY. 2008. Available from: http://www.nature.com/naturebiotechnology

20. Cook NR. Quantifying the added value of new biomarkers: how and how not. Diagn Progn Res. 2018 Dec 11;2(1):14.

21. Rossi R, Murari A, Gaudio P, Gelfusa M. Upgrading Model Selection Criteria with Goodness of Fit Tests for Practical Applications. Entropy. 2020 Apr 15;22(4):447.

**Supplementary Figures and Tables**

**Figure S1. Schematic overview of the different steps of the ClustALL approach using the framework with missing values (best viewed in colour)**. First, (A) a dendrogram and its associated depths are computed considering the original dataset with missing values. The original dataset is then (B). imputed 1,000 times with the MICE algorithm. Then, (C) the 1,000 complete datasets for each resulting depth of the dendrogram are reduced using Principal Component Analysis (PCA). The output is an embedding for each possible depth and imputation (Green Panel). Then, stratification is computed considering the combination distance metric, clustering algorithm and each *Embedding* derived from an imputed dataset (purple panel A). The selection of the optimal number of clusters is based on the consensus from cluster internal validation and the mode of the imputed datasets for each corresponding embedding (purple panel B). Afterwards, a distance matrix (*D_mat_*) between individuals is obtained by computing how often two individuals are assigned to the same cluster in each imputation Then, *D_mat_* is used to calculate a final stratification using correlation-based distance and h-clust (purple panel C). The final step follows the ClustALL framework without imputations (red panel).

**Figure S2.** ***Principal Component projections of the ClustALL robust stratifications based on all the input variables.*** (A-E). Low-dimension representation of the robust stratifications after applying the ClustALL framework to the PREDICT cohort. For each one of the 5 robust stratifications identified by ClustALL, the Principal Component Analysis of all the input variables with the stratification is shown. The *x* (Dim1) and *y* (Dim2) axes represent the first and second principal components respectively, which are linear combinations of the original variables.


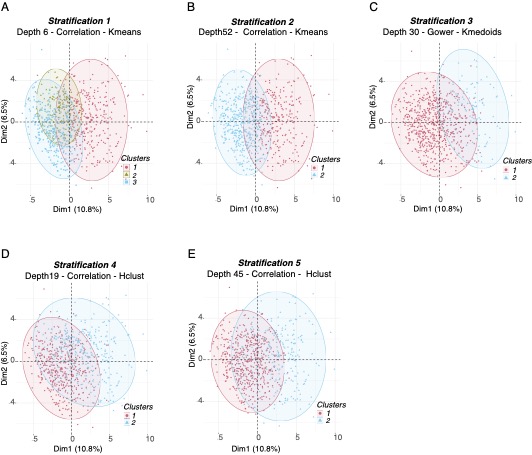


**Figure S3. Frequency Density Plot from bootstrapping the Predict cohort stratification**. The figure displays a bootstrapped density plot used for stratifying the PREDICT data at hospital admission computed within the traditional clustering methods (blue line) versus ClustALL framework (red line), 1,000 times. The x-axis represents the frequency, while the y-axis depicts the resampling times. Traditional methods consider k-means or hierarchical clustering method plus correlation or Gower distances, directly applied to data without considering any dimensionality reduction step.

**Figure S4. Visual representation of the population-based robustness concept**. The figure displays a bootstrapped density plot used for stratifying the PREDICT data at hospital admission computed with ClustALL framework (red line), 1,000 times. The x-axis represents the frequency, while the y-axis depicts the resampling times. The discontinuous lines indicate that more than 85% of the stratifications remain stable despite resampling.

**Figure S5. The window of time between visit and reported event.** Distribution of the window of time (in days) between the reported event and hospital admission visit (in gray) or the reported event and the last reported visit (in blue). Patients with any reported event and at least one visit after hospital admission are included (147). The difference between the window of times is significant (Wilcoxon test p-value <0.001).


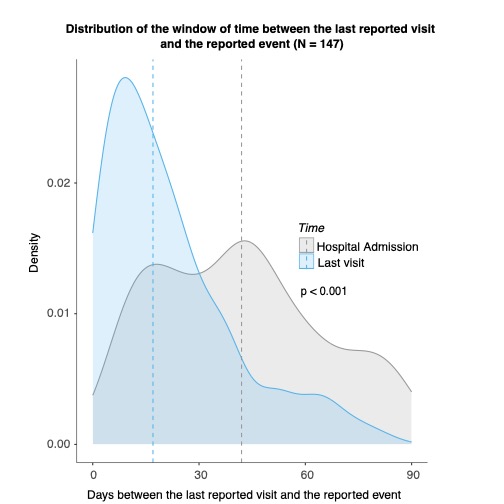


**Table S1. Descriptive statistics of the characteristics for 766 PREDICT patients collected at Hospital Admission**. The values on the left from 1 to 74 indicate the number of variables included as input in the ClustALL framework. The first column represents all the input variables. The second column represents the number and corresponding percentage of missing values per variable. The third to the sixth columns represent the number of patients per variable and corresponding percentage or mean values ± SD for each variable across all patients for the entire cohort, cluster 1, cluster 2 and cluster 3 respectively. The p-values are from the ANOVA and Chi-square analysis for continuous and categorical binary variables between the three clusters.

|  | **Patient Characteristics**  **(PREDICT)** | **Number of missing values** | **Overall population (n=766)** | | **Cluster 1 (n=306)** | **Cluster 2 (n=118)** | | **Cluster 3 (n=342)** | ***P-value*** |
| --- | --- | --- | --- | --- | --- | --- | --- | --- | --- |
|  | **Demographics** |  |  |  | | |  |  |  |
| (1) | Age — years | 0 (0) | 59.44 ± 10.81 | 56.81 ± 10.01 | | | 62.88 ± 11.6 | 60.61 ± 10.7 | <0.01 |
| (2) | Sex — no.(%) | 0 (0) | 247 (32.25) | 103 (33.66) | | | 31 (26.27) | 113 (33.04) | 0.3 |
| (3) | Height — cm | 0 (0) | 170.07 ± 9.21 | 170.54 ± 9.23 | | | 169.65 ± 9.2 | 169.8 ± 9.2 | 0.31 |
| (4) | Weight — kg | 14 (1.83) | 78.4 ± 16.42 | 78.66 ± 17 | | | 77.53 ± 15.55 | 78.46 ± 16.23 | 0.89 |
| (5) | Body Mass Index (kg/m²) | 14 (1.83) | 27.05 ± 5.01 | 26.96 ± 4.98 | | | 26.95 ± 5.19 | 27.17 ± 4.99 | 0.58 |
| (6) | Ethnicity Black or African American — no.(%) | 0 (0) | 2 (0.26) | 0 (0) | | | 2 (1.69) | 0 (0) | <0.01 |
| (7) | Ethnicity Asian — no.(%) | 0 (0) | 12 (1.57) | 8 (2.61) | | | 4 (3.39) | 0 (0) | <0.01 |
| (8) | Ethnicity White — no.(%) | 0 (0) | 740 (96.61) | 295 (96.41) | | | 103 (87.29) | 342 (100) | <0.01 |
| (9) | Ethnicity Other — no.(%) | 0 (0) | 12 (1.57) | 3 (0.98) | | | 9 (7.63) | 0 (0) | <0.01 |
|  | **Cause of cirrhosis** |  |  |  | | |  |  |  |
| (10) | Etiology Alcohol — no.(%) | 0 (0) | 454 (59.27) | 182 (59.48) | | | 70 (59.32) | 202 (59.06) | 0.99 |
| (11) | Etiology Viral — no.(%) | 0 (0) | 83 (10.84) | 32 (10.46) | | | 20 (16.95) | 31 (9.06) | 0.06 |
| (12) | Etiology Alcohol + Viral — no.(%) | 0 (0) | 66 (8.62) | 36 (11.76) | | | 6 (5.08) | 24 (7.02) | 0.03 |
| (13) | Etiology NASH — no.(%) | 0 (0) | 65 (8.49) | 20 (6.54) | | | 10 (8.47) | 35 (10.23) | 0.24 |
| (14) | Etiology Other — no.(%) | 0 (0) | 61 (7.96) | 22 (7.19) | | | 10 (8.47) | 29 (8.48) | 0.81 |
| (15) | Etiology Cryptogenic— no.(%) | 0 (0) | 37 (4.83) | 14 (4.58) | | | 2 (1.69) | 21 (6.14) | 0.15 |
|  | **Main reason for hospitalization** |  |  |  | | |  |  |  |
| (16) | Ascites — no.(%) | 0 (0) | 330 (43.08) | 115 (37.58) | | | 21 (17.8) | 194 (56.73) | <0.01 |
| (17) | Hepatic encephalopathy — no.(%) | 0 (0) | 120 (15.67) | 48 (15.69) | | | 69 (58.47) | 3 (0.88) | <0.01 |
| (18) | Gastrointestinal bleeding — no.(%) | 0 (0) | 116 (15.14) | 24 (7.84) | | | 16 (13.56) | 76 (22.22) | <0.01 |
| (19) | Spontaneous bacterial peritonitis — no.(%) | 0 (0) | 22 (2.87) | 17 (5.56) | | | 0 (0) | 5 (1.46) | <0.01 |
| (20) | Other infection — no.(%) | 0 (0) | 79 (10.31) | 47 (15.36) | | | 6 (5.08) | 26 (7.6) | <0.01 |
|  | **Manifestations at admission** |  |  |  | | |  |  |  |
| (21) | Ascites — no.(%) | 0 (0) | 542 (70.76) | 238 (77.78) | | | 52 (44.07) | 252 (73.68) | <0.01 |
| (22) | Hepatic encephalopathy — no.(%) | 0 (0) | 234 (30.55) | 127 (41.5) | | | 105 (88.98) | 2 (0.58) | <0.01 |
| (23) | Gastrointestinal bleeding — no.(%) | 0 (0) | 108 (14.1) | 24 (7.84) | | | 19 (16.1) | 65 (19.01) | <0.01 |
| (24) | Acute kidney injury — no.(%) | 0 (0) | 45 (5.87) | 27 (8.82) | | | 4 (3.39) | 14 (4.09) | 0.02 |
| (25) | Bacterial Infection — no.(%) | 0 (0) | 222 (28.98) | 139 (45.42) | | | 23 (19.49) | 60 (17.54) | <0.01 |
| (26) | Alcoholic Hepatitis — no.(%) | 0 (0) | 55 (7.18) | 47 (15.36) | | | 0 (0) | 8 (2.34) | <0.01 |
| (27) | Acute Viral Hepatitis — no.(%) | 0 (0) | 5 (0.65) | 3 (0.98) | | | 0 (0) | 2 (0.58) | 0.52 |
| (28) | Hepatocellular Carcinoma — no.(%) | 0 (0) | 34 (4.44) | 12 (3.92) | | | 6 (5.08) | 16 (4.68) | 0.84 |
| (29) | Number of clinical events — number | 0 (0) | 1.63 ± 0.85 | 2.02 ± 0.9 | | | 1.77 ± 0.8 | 1.23 ± 0.59 | <0.01 |
| (30) | Number  of precipitating events — number | 0 (0) | 0.27 ± 0.49 | 0.48 ± 0.59 | | | 0.12 ± 0.32 | 0.13 ± 0.34 | <0.01 |
| (31) | Liver Dysfunction— no.(%) * | 0 (0) | 121 (15.8) | 109 (35.62) | | | 2 (1.69) | 10 (2.92) | <0.01 |
| (32) | Renal Dysfunction — no.(%) * | 0 (0) | 63 (8.22) | 27 (8.82) | | | 9 (7.63) | 27 (7.89) | 0.88 |
| (33) | Brain Dysfunction — no.(%) * | 0 (0) | 215 (28.07) | 119 (38.89) | | | 94 (79.66) | 2 (0.58) | <0.01 |
| (34) | Coagulation Dysfunction — no.(%) * | 0 (0) | 61 (7.96) | 61 (19.93) | | | 0 (0) | 0 (0) | <0.01 |
| (35) | Heart Dysfunction — no.(%) * | 0 (0) | 83 (10.84) | 47 (15.36) | | | 5 (4.24) | 31 (9.06) | <0.01 |
| (36) | Respiratory Dysfunction — no.(%) * | 0 (0) | 31 (4.05) | 20 (6.54) | | | 2 (1.69) | 9 (2.63) | <0.01 |
| (37) | Liver Failure — no.(%) * | 0 (0) | 36 (4.7) | 36 (11.76) | | | 0 (0) | 0 (0) | <0.01 |
| (38) | Brain Liver Failure — no.(%) * | 0 (0) | 19 (2.48) | 8 (2.61) | | | 11 (9.32) | 0 (0) | <0.01 |
| (39) | Coagulation Failure — no.(%) * | 0 (0) | 17 (2.22) | 16 (5.23) | | | 0 (0) | 1 (0.29) | <0.01 |
| (40) | Heart Failure — no.(%) * | 0 (0) | 3 (0.39) | 2 (0.65) | | | 0 (0) | 1 (0.29) | 0.58 |
| (41) | Respiratory Failure — no.(%) * | 0 (0) | 1 (0.13) | 0 (0) | | | 0 (0) | 1 (0.29) | 0.54 |
| (42) | Number of dysfunctions — number | 0 (0) | 0.75 ± 0.86 | 1.25 ± 0.98 | | | 0.95 ± 0.54 | 0.23 ± 0.45 | <0.01 |
| (43) | Number of failures — number | 0 (0) | 0.1 ± 0.3 | 0.2 ± 0.4 | | | 0.09 ± 0.29 | 0.01 ± 0.09 | <0.01 |
|  | **Cirrhosis Severity Scores** |  |  |  | | |  |  |  |
| (44) | CLIF-C-AD | 62 (8.09) | 51.89 ± 7.95 | 56.99 ± 6.58 | | | 48.53 ± 6.4 | 48.48 ± 7.08 | <0.01 |
| (45) | MELD-NA | 0 (0) | 18.61 ± 5.48 | 23.3 ± 3.78 | | | 15.61 ± 3.31 | 15.46 ± 4.25 | <0.01 |
| (46) | MELD | 0 (0) | 15.65 ± 5.01 | 19.92 ± 3.93 | | | 13.29 ± 2.74 | 12.65 ± 3.57 | <0.01 |
| (47) | Child-Pugh | 0 (0) | 2.11 ± 0.58 | 2.54 ± 0.52 | | | 1.92 ± 0.32 | 1.8 ± 0.45 | <0.01 |
| (48) | CLIF-OF | 0 (0) | 6.87 ± 0.98 | 7.57 ± 1.04 | | | 7.06 ± 0.48 | 6.17 ± 0.41 | <0.01 |
|  | **Medical History** |  |  |  | | |  |  |  |
| (49) | Diabetes Mellitus — no.(%) | 0 (0) | 222 (28.98) | 47 (15.36) | | | 60 (50.85) | 115 (33.63) | <0.01 |
| (50) | Arterial hypertension — no.(%) | 0 (0) | 264 (34.46) | 89 (29.08) | | | 48 (40.68) | 127 (37.13) | 0.03 |
| (51) | History of previous decompensations — no.(%) | 13 (1.7) | 631 (82.38) | 239 (78.1) | | | 102 (86.44) | 290 (84.8) | 0.04 |
|  | **Lifestyle** |  |  |  | | |  |  |  |
| (52) | Alcohol consumption in the 3 previous months — no.(%) | 125 (16.32) | 295 (38.51) | 147 (48.04) | | | 21 (17.8) | 127 (37.13) | <0.01 |
| (53) | Active alcohol consumption — no.(%) | 130 (16.97) | 216 (28.19) | 113 (14.75) | | | 14 (18.27) | 89 (11.61) | <0.01 |
| (54) | Active smoker — no.(%) | 2 (0.26) | 423 (55.22) | 167 (54.58) | | | 52 (44.07) | 204 (59.65) | 0.01 |
|  | **Laboratory Variables** |  |  |  | | |  |  |  |
| (55) | Serum Alanine Transaminase — U/L | 17 (2.22) | 37.85 ± 42.4 | 50.81 ± 60.01 | | | 28.65 ± 19.24 | 29.42 ± 20.83 | <0.01 |
| (56) | Serum Aspartate aminotransferase — U/L | 52 (6.79) | 70.38 ± 72.32 | 96.41 ± 93.01 | | | 47.66 ± 29.42 | 54.94 ± 51.7 | <0.01 |
| (57) | Serum Albumin — g/dL | 45 (5.87) | 2.9 ± 0.59 | 2.7 ± 0.52 | | | 2.96 ± 0.56 | 3.06 ± 0.61 | <0.01 |
| (58) | Serum Total Bilirubin — mg/dL | 0 (0) | 4.05 ± 4.36 | 7.02 ± 5.4 | | | 2.13 ± 1.49 | 2.06 ± 1.62 | <0.01 |
| (59) | Serum Gamma-glutamyl transferase — U/L | 94 (12.27) | 170.57 ± 244.25 | 182.6 ± 232.68 | | | 92.91 ± 96.48 | 186.6 ± 282.38 | 0.77 |
| (60) | Serum C-Reactive Protein — mg/L | 85 (11.1) | 27.28 ± 35.09 | 37.83 ± 38.85 | | | 15.96 ± 23.08 | 21.74 ± 32.47 | <0.01 |
| (61) | Serum Sodium— mEq/L | 0 (0) | 135.26 ± 5.28 | 133.33 ± 5.5 | | | 137.02 ± 4.51 | 136.39 ± 4.77 | <0.01 |
| (62) | Serum Potassium — mEq/L | 8 (1.04) | 4.04 ± 0.64 | 4 ± 0.72 | | | 4.06 ± 0.56 | 4.08 ± 0.59 | 0.13 |
| (63) | Serum Glucose — mg/dL | 132 (17.23) | 124.03 ± 51.33 | 119.04 ± 44.33 | | | 136.51 ± 54.15 | 124.19 ± 55.41 | 0.23 |
| (64) | Hemoglobin — g/dL | 1 (0.13) | 10.39 ± 2.23 | 10.42 ± 2.2 | | | 10.5 ± 2.11 | 10.32 ± 2.29 | 0.53 |
| (65) | Hematocrit — % | 14 (1.83) | 30.76 ± 6.17 | 30.43 ± 6.14 | | | 31.16 ± 5.95 | 30.91 ± 6.27 | 0.33 |
| (66) | Creatinine — mg/dL | 0 (0) | 0.95 ± 0.34 | 0.94 ± 0.36 | | | 0.99 ± 0.35 | 0.96 ± 0.33 | 0.45 |
| (67) | White blood cell count — x10^9/L | 0 (0) | 6.97 ± 4.08 | 8.87 ± 4.72 | | | 5.09 ± 2.06 | 5.91 ± 3.22 | <0.01 |
| (68) | Lymphocytes — x10^9/L | 145 (18.93) | 1.13 ± 0.61 | 1.26 ± 0.65 | | | 1.09 ± 0.63 | 1.02 ± 0.55 | <0.01 |
| (69) | Monocytes— x10^9/L | 157 (20.5) | 0.67 ± 0.38 | 0.84 ± 0.41 | | | 0.54 ± 0.29 | 0.56 ± 0.3 | <0.01 |
| (70) | Neutrophils— x10^9/L | 133 (17.36) | 4.76 ± 3.46 | 6.29 ± 4.13 | | | 3.16 ± 1.58 | 3.95 ± 2.66 | <0.01 |
| (71) | INR (International Normalized Ratio) | 0 (0) | 1.52 ± 0.48 | 1.79 ± 0.63 | | | 1.36 ± 0.19 | 1.34 ± 0.22 | <0.01 |
| (72) | Platelet — x10^3/µL | 7 (0.91) | 115.22 ± 75.35 | 114.89 ± 80.93 | | | 99.14 ± 60.59 | 121.06 ± 74.14 | 0.27 |
| (73) | SpO2 — (%) | 48 (6.27) | 97 ± 2.12 | 96.82 ± 2.27 | | | 97 ± 2.17 | 97.17 ± 1.95 | 0.04 |
| (74) | SpO2/FiO2 Ratio | 48 (6.27) | 456.27 ± 30.66 | 452.45 ± 37.31 | | | 459.07 ± 25.23 | 458.71 ± 24.97 | 0.01 |
|  | **Outcomes** |  |  |  | | |  |  |  |
|  | 28-day mortality — no.(%) | 0 (0) | 34 (4.44) | 23 (3) | | | 3 (0.39) | 8 (1.04) | <0.01 |
|  | 90-day mortality — no.(%) | 0 (0) | 103 (13.45) | 62 (8.09) | | | 5 (0.65) | 36 (4.70) | <0.01 |
|  | 28-day ACLF development — no.(%) | 0 (0) | 64 (8.36) | 43 (5.61) | | | 6 (0.78) | 15 (1.96) | <0.01 |
|  | 90-day ACLF development — no.(%) | 0 (0) | 114 (14.88) | 70 (9.14) | | | 10 (1.31) | 34 (4.44) | <0.01 |
|  | 28-day transplant — no.(%) | 0 (0) | 17 (2.22) | 13 (1.70) | | | 1 (0.13) | 3 (0.39) | <0.01 |
|  | 90-day transplant — no.(%) | 0 (0) | 49 (6.40) | 35 (4.57) | | | 2 (0.26) | 12 (1.57) | <0.01 |
|  | *ACLF=Acute-on-chronic liver failure, FiO2= Fraction of inspired oxygen NASH= Non-alcoholic steatohepatitis,*  *SpO2= Saturation of peripheral Oxygen.* | | | | | | | | |
|  | ** as defined in Jalan, R., et al. (2014). Development and validation of a prognostic score to predict mortality in patients with acute-on-chronic liver failure. Journal of Hepatology, 61(5), 1038–1047. https://doi.org/10.1016/j.jhep.2014.06.012* | | | | | | | | |

**Table S2. Patient distribution through the clusters when using the classical clustering methodologies**. The distribution of the PREDICT patients after applying different distance metrics (Correlation and Gower) and clustering techniques (K-means, Hierarchical Clustering, and K-medoids) considering 2 or 3 clusters are shown.

| ***Correlation K-means*** | |  |  |
| --- | --- | --- | --- |
| ***k=2*** | *Cluster 1 =* 734 | *Cluster 2 =* 32 |  |
| ***k=3*** | *Cluster 1 =* 732 | *Cluster 2 =* 25 | *Cluster 3 =* 9 |
|  |  |  |  |
| ***Correlation Hierarchical Clustering*** | | |  |
| ***k=2*** | *Cluster 1 =* 757 | *Cluster 2 =* 9 |  |
| ***k=3*** | *Cluster 1 =* 734 | *Cluster 2 =* 23 | *Cluster 3 =* 9 |
|  |  |  |  |
| ***Gower K-medoids*** | |  |  |
| ***k=2*** | *Cluster 1 =* 512 | *Cluster 2 =* 254 |  |
| ***k=3*** | *Cluster 1 =* 321 | *Cluster 2 =* 232 | *Cluster 3 =* 213 |
|  |  |  |  |
| ***Gower Hierarchical Clustering*** | | |  |
| ***k=2*** | *Cluster 1 =* 655 | *Cluster 2* = 111 |  |
| ***k=3*** | *Cluster 1 =* 360 | *Cluster 2* = 295 | *Cluster 3 =* 111 |
|  |  |  |  |

**Table S3-S7. Summary of the most predictive variables in the identified clusters for 1 to 5 Stratification representatives** **in PREDICT cohort at hospital admission.** The p-values are derived from the ANOVA analysis for continuous variables or from χ2 analysis for binary and categorical variables. Values are the mean ± SD unless indicated otherwise.

**Table S3.** **Stratification 1** (Height 6, Correlation, K-means).

| **Clinical Feature** | **Cluster 1 (n=306)** | **Cluster 2 (n=118)** | **Cluster 3 (n=342)** | ***P-value*** |
| --- | --- | --- | --- | --- |
| Diabetes Mellitus — no.(%) | 47 (15.36) | 60 (50.85) | 115 (33.63) | 1.90E-13 |
| Hepatic Encephalopathy — no.(%) | 127 (41.5) | 105 (88.98) | 2 (0.58) | 3.77E-77 |
| Number of clinical events — number | 2.02 ± 0.9 | 1.77 ± 0.8 | 1.23 ± 0.59 | 1.17495E-35 |
| Organ Dysfunction — number | 1.25 ± 0.98 | 0.95 ± 0.54 | 0.23 ± 0.45 | 9.42939E-61 |
| Number of precipitating events — number | 0.48 ± 0.59 | 0.12 ± 0.32 | 0.13 ± 0.34 | 1.04935E-22 |
| Serum Albumin — g/dL | 2.7 ± 0.52 | 2.96 ± 0.56 | 3.06 ± 0.61 | 6.64445E-15 |
| Serum Aspartate Aminotransferase — units/L | 96.41 ± 93.01 | 47.66 ± 29.42 | 54.94 ± 51.7 | 6.87725E-16 |
| Serum CRP — mg/L | 37.83 ± 38.85 | 15.96 ± 23.08 | 21.74 ± 32.47 | 1.46111E-11 |
| Serum Bilirubin —mg/dL | 7.02 ± 5.4 | 2.13 ± 1.49 | 2.06 ± 1.62 | 7.86805E-62 |
| INR | 1.79 ± 0.63 | 1.36 ± 0.19 | 1.34 ± 0.22 | 5.42777E-39 |
| Serum Sodium — mEq/L | 133.33 ± 5.5 | 137.02 ± 4.51 | 136.39 ± 4.77 | 1.6674E-16 |
| White Blood Cells — x10^3^/mm^3^ | 8.87 ± 4.72 | 5.09 ± 2.06 | 5.91 ± 3.22 | 1.27361E-27 |
| **CRP=C-Reactive Protein, INR= International Normalized Ratio.* | | | | |
| *¶ Counting the hospital admission visit.* |  |  |  |  |

**Table S4.** Stratification 2 *(Height 30, Correlation, K-means).*

| **Clinical Feature** | **Cluster 1 (n=283)** | **Cluster 2 (n=483)** | ***P-value*** |
| --- | --- | --- | --- |
| Diabetes Mellitus — no.(%) | 45 (15.9) | 177 (36.65) | 1.68E-09 |
| Gastrointestinal Bleeding — no.(%) | 20 (7.07) | 88 (18.22) | 3.00E-05 |
| Number of clinical events — number | 2.04 ± 0.9 | 1.38 ± 0.71 | 1.84E-23 |
| Respiratory Dysfunction — no.(%) | 23 (8.13) | 8 (1.66) | 2.71E-05 |
| Organ Dysfunction — number | 1.28 ± 0.99 | 0.44 ± 0.59 | 3.58E-36 |
| Number of precipitating events — number | 0.51 ± 0.6 | 0.13 ± 0.33 | 3.84E-24 |
| Serum Albumin — g/dL | 2.7 ± 0.52 | 3.02 ± 0.6 | 5.29E-13 |
| CRP — mg/L | 39.2 ± 39.7 | 20.3 ± 30 | 2.58E-13 |
| Serum Bilirubin—mg/dL | 7.33 ± 5.5 | 2.13 ± 1.58 | 1.04E-68 |
| INR | 1.8 ± 0.65 | 1.36 ± 0.23 | 1.01E-38 |
| Serum Sodium — mEq/L | 133.2 ± 5.44 | 136.47 ± 4.79 | 2.16E-17 |
| White Blood Cells — x10^3^/mm^3^ | 8.93 ± 4.79 | 5.82 ± 3.07 | 4.43E-26 |
| **CRP=C-Reactive Protein, INR= International Normalized Ratio.* | | | |
| *¶ Counting the hospital admission visit.* |  |  |  |

**Table S5.** Stratification 3 (*Height 30, Gower, H-clust).*

| **Clinical Feature** | **Cluster 1 (n=666)** | **Cluster 2 (n=100)** | ***P-value*** |
| --- | --- | --- | --- |
| Number of clinical events — number | 1.53 ± 0.78 | 2.27 ± 0.99 | 6.47E-17 |
| Liver Dysfunction — no.(%) | 59 (8.86) | 62 (62) | 3.55E-41 |
| Cerebral Dysfunction — no.(%) | 168 (25.23) | 47 (47) | 1.09E-05 |
| Coagulation Dysfunction — no.(%) | 0 (0) | 61 (61) | 3.39E-96 |
| Number of dysfunctions — number | 0.56 ± 0.66 | 1.99 ± 1.02 | 2.68E-60 |
| Liver Failure — no.(%) | 23 (3.45) | 13 (13) | 7.72E-05 |
| INR | 1.44 ± 0.32 | 2.08 ± 0.86 | 2.41E-39 |
| Serum Sodium — mEq/L | 135.59 ± 5.1 | 133.1 ± 5.92 | 1.01E-05 |
| **INR= International Normalized Ratio .* | | | |
| *¶ Counting the hospital admission visit.* |  |  |  |

**Table S6.** Stratification 4 (*Height* 19, Correlation, *H-clust*)

| **Clinical Feature** | **Cluster 1 (n=431)** | **Cluster 2 (n=335)** | ***P-value*** |
| --- | --- | --- | --- |
| Age — number | 60.19 ± 10.6 | 58.48 ± 11.01 | 3.00E-02 |
| Hepatic encephalopathy — no.(%) | 34 (7.89) | 200 (59.7) | 2.83E-53 |
| Gastrointestinal Bleeding — no.(%) | 81 (18.79) | 27 (8.06) | 3.63E-05 |
| Acute Viral Hepatitis — no.(%) | 5 (1.16) | 0 (0) | 1.27E-01 |
| Liver Dysfunction — no.(%) | 30 (6.96) | 91 (27.16) | 6.11E-14 |
| Coagulation Dysfunction — no.(%) | 2 (0.46) | 59 (17.61) | 1.11E-17 |
| Respiratory Dysfunction — no.(%) | 0 (0) | 31 (9.25) | 3.79E-10 |
| Number of failures — number | 0 ± 0.05 | 0.22 ± 0.42 | 8.89E-24 |
| *¶ Counting the hospital admission visit.* | |  |  |
|  |  |  |  |

**Table S7.** *Stratification 5* (*Height* 45, Correlation, *H-clust*).

| **Clinical Feature** | **Cluster 1 (n=522)** | **Cluster 2 (n=244)** | ***P-value*** |
| --- | --- | --- | --- |
| Alcohol etiology — no.(%) | 295 (56.51) | 159 (65.16) | 2.84E-02 |
| Gastrointestinal Bleeding — no.(%) | 94 (18.01) | 14 (5.74) | 9.21E-06 |
| Acute Alcoholic-steatohepatitis — no.(%) | 6 (1.15) | 49 (20.08) | 1.32E-20 |
| Hepatocellular carcinoma — no.(%) | 29 (5.56) | 5 (2.05) | 4.47E-02 |
| Liver Dysfunction — no.(%) | 25 (4.79) | 96 (39.34) | 9.21E-34 |
| Coagulation Dysfunction — no.(%) | 3 (0.57) | 58 (23.77) | 1.09E-27 |
| Respiratory Dysfunction — no.(%) | 4 (0.77) | 27 (11.07) | 6.04E-11 |
| Number of failures — number | 0.01 ± 0.09 | 0.3 ± 0.46 | 1.35E-34 |
| Number of precipitating events — number | 0.17 ± 0.38 | 0.48 ± 0.6 | 2.18E-16 |
| Serum Bilirubin —mg/dl | 2.46 ± 1.91 | 7.45 ± 5.92 | 1.14E-57 |
| Serum Sodium — mEq/L | 136.04 ± 5.04 | 133.6 ± 5.39 | 1.54E-09 |
| Smoker — no.(%) | 304 (58.24) | 119 (48.77) | 1.75E-02 |
| *¶ Counting the inclusion visit.* | |  |  |
|  |  |  |  |

**Table S8.** Distribution of the *AD-strat* clustering-based labelling in PREDICT cohort at hospital admission obtained with ClustALL vs. acute decompensation of cirrhosis classification as defined in The PREDICT study from Trebicka, J, et al.

| N =766 | SDC | UDC | pre-ACLF |
| --- | --- | --- | --- |
| Cluster 1 — no.(%) | 157 (33.7) | 78 (42.4) | 71 (61.2) |
| Cluster 2 — no.(%) | 83 (17.8) | 25 (13.6) | 10 (8.6) |
| Cluster 3 — no.(%) | 226 (48.5) | 81 (44) | 35 (30.2) |

**pre-ACLF= pre-acute-on-chronic liver failure, SDC=Stable decompensated cirrhosis, UDC= Unstable decompensated cirrhosis.*

**Table S9. Characteristics for 580 ACLARA patients collected at Hospital Admission**. The first column represents the ACLARA variables. The second column represents the number and corresponding percentage of missing values per variable. The third to the sixth columns represent the number of patients per variable and corresponding percentage or mean values ± SD for each variable across all patients for the entire cohort, cluster 1, cluster 2 and cluster 3 respectively. The p-values are from the ANOVA and Chi-square analysis for continuous and categorical binary variables between the three clusters.

| **Patient Characteristics (ACLARA)** | **Number of missing values** | | **Overall population (n=580)** | | **Cluster 1 (n=185)** | | **Cluster 2 (n=99)** | | **Cluster 3 (n=296)** | | ***P-value*** | |  |  |  |
| --- | --- | --- | --- | --- | --- | --- | --- | --- | --- | --- | --- | --- | --- | --- | --- |
| **Demographics** |  | |  | |  | |  | |  | |  | |  |  |  |
| Age — years | 0 (0) | | 58.1 ± 12.3 | | 54.08 ± 12.44 | | 62.74 ± 10.04 | | 59.06 ± 12.19 | | <0.01 | |  |  |  |
| Sex — no.(%) | 0 (0) | | 205 (35.34) | | 50 (27.03) | | 33 (33.33) | | 122 (41.22) | | <0.01 | |  |  |  |
| Body Mass Index (kg/m²) | 103 (17.76) | | 27.11 ± 4.76 | | 27.21 ± 4.93 | | 27.26 ± 4.67 | | 27.01 ± 4.7 | | 0.63 | |  |  |  |
| Ethnicity Black or African American — no.(%) | 0 (0) | | 105 (18.1) | | 38 (20.54) | | 21 (21.21) | | 46 (15.54) | | 0.26 | |  |  |  |
| Ethnicity Asian — no.(%) | 0 (0) | | 1 (0.17) | | 0 (0) | | 0 (0) | | 1 (0.34) | | 0.62 | |  |  |  |
| Ethnicity Indian or Alaska Native American— no.(%) | 0 (0) | | 98 (16.9) | | 40 (21.62) | | 15 (15.15) | | 43 (14.53) | | 0.11 | |  |  |  |
| Ethnicity White — no.(%) | 0 (0) | | 301 (51.9) | | 82 (44.32) | | 53 (53.54) | | 166 (56.08) | | 0.12 | |  |  |  |
| Multiple — no.(%) | 0 (0) | | 75 (12.93) | | 25 (13.51) | | 10 (10.1) | | 40 (13.51) | | 0.65 | |  |  |  |
| **Cause of cirrhosis** |  | |  | |  | |  | |  | |  | |  |  |  |
| Etiology Alcohol — no.(%) | 0 (0) | | 208 (35.86) | | 60 (32.43) | | 36 (36.36) | | 112 (37.84) | | 0.48 | |  |  |  |
| Etiology Viral — no.(%) | 0 (0) | | 77 (13.28) | | 23 (12.43) | | 14 (14.14) | | 40 (13.51) | | 0.91 | |  |  |  |
| Etiology Alcohol + Viral — no.(%) | 0 (0) | | 35 (6.03) | | 10 (5.41) | | 5 (5.05) | | 20 (6.76) | | 0.75 | |  |  |  |
| Etiology NASH — no.(%) | 0 (0) | | 102 (17.59) | | 35 (18.92) | | 18 (18.18) | | 49 (16.55) | | 0.79 | |  |  |  |
| Etiology Other — no.(%) | 0 (0) | | 66 (11.38) | | 18 (9.73) | | 12 (12.12) | | 36 (12.16) | | 0.69 | |  |  |  |
| Etiology Cryptogenic— no.(%) | 0 (0) | | 92 (15.86) | | 39 (21.08) | | 14 (14.14) | | 39 (13.18) | | 0.06 | |  |  |  |
| **Main reason for hospitalization** |  | |  | |  | |  | |  | |  | |  |  |  |
| Ascites — no.(%) | 0 (0) | | 138 (23.79) | | 40 (21.62) | | 12 (12.12) | | 86 (29.05) | | <0.01 | |  |  |  |
| Hepatic encephalopathy — no.(%) | 0 (0) | | 109 (18.79) | | 50 (27.03) | | 53 (53.54) | | 6 (2.03) | | <0.01 | |  |  |  |
| Gastrointestinal bleeding — no.(%) | 0 (0) | | 175 (30.17) | | 28 (15.14) | | 20 (20.2) | | 127 (42.91) | | <0.01 | |  |  |  |
| Spontaneous bacterial peritonitis — no.(%) | 0 (0) | | 51 (8.79) | | 24 (12.97) | | 3 (3.03) | | 24 (8.11) | | 0.02 | |  |  |  |
| Other infection — no.(%) | 0 (0) | | 83 (14.31) | | 32 (17.3) | | 8 (8.08) | | 43 (14.53) | | 0.11 | |  |  |  |
| **Manifestations at admission** |  | |  | |  | |  | |  | |  | |  |  |  |
| Ascites — no.(%) | 0 (0) | | 365 (62.93) | | 145 (78.38) | | 48 (48.48) | | 172 (58.11) | | <0.01 | |  |  |  |
| Hepatic encephalopathy — no.(%) | 0 (0) | | 189 (32.59) | | 90 (48.65) | | 99 (100) | | 0 (0) | | <0.01 | |  |  |  |
| Gastrointestinal bleeding — no.(%) | 0 (0) | | 183 (31.55) | | 33 (17.84) | | 21 (21.21) | | 129 (43.58) | | <0.01 | |  |  |  |
| Acute kidney injury — no.(%) | 0 (0) | | 87 (15) | | 37 (20) | | 8 (8.08) | | 42 (14.19) | | 0.02 | |  |  |  |
| Bacterial Infection — no.(%) | 0 (0) | | 242 (41.72) | | 108 (58.38) | | 33 (33.33) | | 101 (34.12) | | <0.01 | |  |  |  |
| Alcoholic Hepatitis — no.(%) | 0 (0) | | 122 (21.03) | | 75 (40.54) | | 11 (11.11) | | 36 (12.16) | | <0.01 | |  |  |  |
| Acute Viral Hepatitis — no.(%) | 0 (0) | | 8 (1.38) | | 3 (1.62) | | 1 (1.01) | | 4 (1.35) | | 0.91 | |  |  |  |
| Hepatocellular Carcinoma — no.(%) | 0 (0) | | 18 (3.1) | | 8 (4.32) | | 4 (4.04) | | 6 (2.03) | | 0.31 | |  |  |  |
| Number of clinical events — number | 0 (0) | | 2.09 ± 0.97 | | 2.7 ± 1 | | 2.27 ± 0.81 | | 1.66 ± 0.75 | | <0.01 | |  |  |  |
| Number  of precipitating events — number | 0 (0) | | 0.42 ± 0.57 | | 0.76 ± 0.63 | | 0.21 ± 0.41 | | 0.28 ± 0.47 | | <0.01 | |  |  |  |
| Liver Dysfunction— no.(%) * | 0 (0) | | 72 (12.41) | | 56 (30.27) | | 5 (5.05) | | 11 (3.72) | | <0.01 | |  |  |  |
| Renal Dysfunction — no.(%) * | 0 (0) | | 61 (10.52) | | 26 (14.05) | | 12 (12.12) | | 23 (7.77) | | 0.08 | |  |  |  |
| Brain Dysfunction — no.(%) * | 0 (0) | | 174 (30) | | 81 (43.78) | | 93 (93.94) | | 0 (0) | | <0.01 | |  |  |  |
| Coagulation Dysfunction — no.(%) * | 0 (0) | | 58 (10) | | 45 (24.32) | | 6 (6.06) | | 7 (2.36) | | <0.01 | |  |  |  |
| Heart Dysfunction — no.(%) * | 0 (0) | | 52 (8.97) | | 24 (12.97) | | 5 (5.05) | | 23 (7.77) | | 0.05 | |  |  |  |
| Respiratory Dysfunction — no.(%) * | 0 (0) | | 31 (5.34) | | 16 (8.65) | | 5 (5.05) | | 10 (3.38) | | 0.04 | |  |  |  |
| Liver Failure — no.(%) * | 0 (0) | | 9 (1.55) | | 8 (4.32) | | 0 (0) | | 1 (0.34) | | <0.01 | |  |  |  |
| Brain Liver Failure — no.(%) * | 0 (0) | | 15 (2.59) | | 9 (4.86) | | 6 (6.06) | | 0 (0) | | <0.01 | |  |  |  |
| Coagulation Failure — no.(%) * | 0 (0) | | 10 (1.72) | | 7 (3.78) | | 0 (0) | | 3 (1.01) | | 0.03 | |  |  |  |
| Heart Failure — no.(%) * | 0 (0) | | 3 (0.52) | | 2 (1.08) | | 0 (0) | | 1 (0.34) | | 0.40 | |  |  |  |
| Respiratory Failure — no.(%) * | 0 (0) | | 3 (0.52) | | 0 (0) | | 0 (0) | | 3 (1.01) | | 0.24 | |  |  |  |
| Number of dysfunctions — number | 0 (0) | | 0.77 ± 0.89 | | 1.34 ± 1 | | 1.27 ± 0.59 | | 0.25 ± 0.49 | | 0.00 | |  |  |  |
| Number of failures — number | 0 (0) | | 0.07 ± 0.25 | | 0.14 ± 0.35 | | 0.06 ± 0.24 | | 0.03 ± 0.16 | | <0.01 | |  |  |  |
| **Cirrhosis Severity Scores** |  | |  | |  | |  | |  | |  | |  |  |  |
| Child-Pugh | 12 (2.07) | | 8.58 ± 1.97 | | 10.18 ± 1.37 | | 8.8 ± 1.74 | | 7.5 ± 1.62 | | <0.01 | |  |  |  |
| CLIF-C-AD | 20 (3.45) | | 50.65 ± 9.03 | | 54.6 ± 8.92 | | 51.19 ± 8.25 | | 48 ± 8.43 | | <0.01 | |  |  |  |
| CLIF-OF | 0 (0) | | 6.81 ± 0.97 | | 7.48 ± 1.07 | | 7.27 ± 0.68 | | 6.23 ± 0.52 | | <0.01 | |  |  |  |
| MELD | 1 (0.17) | | 15.31 ± 4.86 | | 19.48 ± 3.9 | | 14.56 ± 4.14 | | 12.95 ± 3.8 | | <0.01 | |  |  |  |
| MELD-Na | 3 (0.52) | | 18.08 ± 5.51 | | 22.56 ± 4.09 | | 17.51 ± 4.95 | | 15.47 ± 4.64 | | <0.01 | |  |  |  |
| **Medical History** |  | |  | |  | |  | |  | |  | |  |  |  |
| Diabetes Mellitus — no.(%) | 0 (0) | | 228 (39.31) | | 12 (6.49) | | 63 (63.64) | | 153 (51.69) | | <0.01 | |  |  |  |
| Arterial hypertension — no.(%) | 0 (0) | | 193 (33.28) | | 38 (20.54) | | 47 (47.47) | | 108 (36.49) | | <0.01 | |  |  |  |
| History of previous decompensations — no.(%) | 12 (2.07) | | 494 (85.17) | | 161 (87.03) | | 90 (90.91) | | 243 (82.09) | | 0.07 | |  |  |  |
| **Lifestyle** |  | |  | |  | |  | |  | |  | |  |  |  |
| Alcohol consumption in the 3 previous months — no.(%) | 0 (0) | | 224 (38.62) | | 83 (44.86) | | 35 (35.35) | | 106 (35.81) | | 0.11 | |  |  |  |
| Active smoker — no.(%) | 0 (0) | | 195 (33.62) | | 67 (36.22) | | 26 (26.26) | | 102 (34.46) | | 0.22 | |  |  |  |
| **Laboratory Variables** |  | |  | |  | |  | |  | |  | |  |  |  |
| Serum Alanine Transaminase — U/L | 20 (3.45) | | 43.8 ± 48.17 | | 51.25 ± 54.79 | | 39.04 ± 42.45 | | 40.74 ± 45.06 | | 0.03 | |  |  |  |
| Serum Aspartate aminotransferase — U/L | 22 (3.79) | | 67.5 ± 64.76 | | 90.85 ± 93.03 | | 53.71 ± 27.65 | | 57.52 ± 45.54 | | <0.01 | |  |  |  |
| Serum Albumin — g/dL | 12 (2.07) | | 2.92 ± 0.58 | | 2.71 ± 0.52 | | 2.94 ± 0.6 | | 3.05 ± 0.57 | | <0.01 | |  |  |  |
| Serum Total Bilirubin — mg/dL | 1 (0.17) | | 3.09 ± 3.25 | | 5.29 ± 4.19 | | 2.37 ± 2.08 | | 1.96 ± 1.96 | | <0.01 | |  |  |  |
| Serum Gamma-glutamyl transferase — U/L | 136 (23.45) | | 143.55 ± 175.46 | | 155.94 ± 222.34 | | 123.37 ± 123.06 | | 142.55 ± 155.96 | | 0.49 | |  |  |  |
| Serum C-Reactive Protein — mg/L | 36 (6.21) | | 35.45 ± 38.44 | | 41.7 ± 40.04 | | 32 ± 42.65 | | 32.69 ± 35.49 | | 0.02 | |  |  |  |
| Serum Sodium— mEq/L | 2 (0.34) | | 135.7 ± 5.45 | | 134.03 ± 5.77 | | 135.72 ± 6.36 | | 136.74 ± 4.62 | | <0.01 | |  |  |  |
| Serum Potassium — mEq/L | 6 (1.03) | | 4.19 ± 0.61 | | 4.19 ± 0.68 | | 4.2 ± 0.56 | | 4.18 ± 0.57 | | 0.74 | |  |  |  |
| Hemoglobin — g/dL | 3 (0.52) | | 9.91 ± 2.19 | | 10.09 ± 2.1 | | 10.2 ± 2.08 | | 9.7 ± 2.27 | | 0.04 | |  |  |  |
| Hematocrit — % | 4 (0.69) | | 29.56 ± 6.14 | | 29.74 ± 6.1 | | 30.28 ± 5.91 | | 29.2 ± 6.23 | | 0.29 | |  |  |  |
| Creatinine — mg/dL | 0 (0) | | 0.98 ± 0.36 | | 1.03 ± 0.39 | | 0.97 ± 0.35 | | 0.94 ± 0.33 | | 0.01 | |  |  |  |
| White blood cell count — x10^9/L | 18 (3.1) | | 6.08 ± 3.83 | | 7.25 ± 4.64 | | 5.39 ± 2.67 | | 5.57 ± 3.44 | | <0.01 | |  |  |  |
| Lymphocytes — x10^9/L | 129 (22.24) | | 0.99 ± 0.73 | | 1.09 ± 0.89 | | 0.92 ± 0.48 | | 0.96 ± 0.69 | | 0.07 | |  |  |  |
| Monocytes — x10^9/L | 165 (28.45) | | 0.55 ± 0.42 | | 0.62 ± 0.4 | | 0.5 ± 0.24 | | 0.52 ± 0.48 | | 0.01 | |  |  |  |
| Neutrophils — x10^9/L | 126 (21.72) | | 4.28 ± 3.21 | | 5.29 ± 4.04 | | 3.74 ± 2.26 | | 3.83 ± 2.73 | | <0.01 | |  |  |  |
| INR (International Normalized Ratio) | 0 (0) | | 1.56 ± 0.39 | | 1.81 ± 0.45 | | 1.53 ± 0.31 | | 1.42 ± 0.29 | | <0.01 | |  |  |  |
| Platelet — x10^3/µL | 5 (0.86) | | 104.22 ± 66.73 | | 99.62 ± 62.91 | | 89.15 ± 45.35 | | 112.14 ± 73.73 | | 0.03 | |  |  |  |
| SpO2 — (%) | 15 (2.59) | | 96.24 ± 2.32 | | 96.3 ± 2.23 | | 96.2 ± 2.45 | | 96.22 ± 2.34 | | 0.73 | |  |  |  |
| SpO2/FiO2 Ratio | 15 (2.59) | | 449.3 ± 39.73 | | 447.5 ± 42.24 | | 447.46 ± 44.5 | | 451.03 ± 36.34 | | 0.32 | |  |  |  |
| **Outcomes** |  | |  | |  | |  | |  | |  | |  |  |  |
| 28-day mortality — no.(%) | 0 (0) | | 59 (10.2) | | 26 (4.48%) | | 13 (2.24) | | 20 (3.45) | | 0.02 | |  |  |  |
| 28-day ACLF development — no.(%) | 0 (0) | | 65 (11.2) | | 29 (5%) | | 16 (2.76) | | 20 (3.45) | | <0.01 | |  |  |  |
| 28-day transplant — no.(%) | 0 (0) | | 18 (3.1) | | 12 (2.07%) | | 3 (0.52) | | 3 (0.52) | | <0.01 | |  |  |  |
| *ACLF=Acute-on-chronic liver failure, FiO2= Fraction of inspired oxygen NASH= Non-alcoholic steatohepatitis,*  *SpO2= Saturation of peripheral Oxygen..* | | | | | | | | | | | | | |  |  |
| ** as defined in Jalan, R., et al. (2014). Development and validation of a prognostic score to predict mortality in patients with acute-on-chronic liver failure. Journal of Hepatology, 61(5), 1038–1047. https://doi.org/10.1016/j.jhep.2014.06.012* | | | | | | | | | | | | | | |  |
|  | |  | |  | |  | |  | |  | |  | | | |

**Table S10**. **Performance measures of kNN classifier.** Summary of the evaluated metrics used to set the choice of the *k* in the kNN model for label transfer.

| **k** | **Accuracy** | **AUC** | **FP** | **FN** | **ER** |
| --- | --- | --- | --- | --- | --- |
| 25 | 0.8304348 | 0.6782492 | 3 | 4 | 0.0304348 |
| 26 | 0.8304348 | 0.6819865 | 3. | 4 | 0.0304348 |
| 27 | 0.8391304 | 0.6814254 | 4 | 4 | 0.0347826 |
| 28 | 0.8304348 | 0.6853535 | 5 | 2 | 0.0304348 |
| 29 | 0.826087 | 0.6886756 | 5 | 3 | 0.0347826 |
| 30 | 0.826087 | 0.6884736 | 4 | 3 | 0.0304348 |
| *AUC=Area Under the Curve, FP=False Positives, FN=False Negatives, ER=Error Rate* | | | | | |

**Table S11. Summary of the most predictive variables in the identified clusters in ACLARA cohort at hospital admission.** The p-values are derived from the ANOVA analysis for continuous variables or from χ2 analysis for binary and categorical variables. Values are the mean ± SD unless indicated otherwise.

| **Clinical Feature** | **Cluster 1 (n=185)** | **Cluster 2 (n=99)** | **Cluster 3 (n=296)** | **P-value** |
| --- | --- | --- | --- | --- |
| Diabetes Mellitus — no.(%) | 12 (6.49) | 63 (63.64) | 153 (51.69) | 2.50E-28 |
| Hepatic Encephalophaty — no.(%) | 90 (48.65) | 99 (100) | 0 (0) | 5.48E-81 |
| Clinical Events — number | 2.7 ± 1 | 2.27 ± 0.81 | 1.66 ± 0.75 | 1.98E-27 |
| Organ Dysfunction — number | 1.34 ± 1 | 1.27 ± 0.59 | 0.25 ± 0.49 | 1.35E-56 |
| Precipitating Events — number | 0.76 ± 0.63 | 0.21 ± 0.41 | 0.28 ± 0.47 | 2.42E-20 |
| Serum Albumine — g/dL | 2.71 ± 0.52 | 2.94 ± 0.6 | 3.05 ± 0.57 | 1.80E-10 |
| Serum Aspartate Aminotransferase — units/L | 90.85 ± 93.03 | 53.71 ± 27.65 | 57.52 ± 45.54 | 1.20E-07 |
| Serum CRP — mg/L | 41.7 ± 40.04 | 32 ± 42.65 | 32.69 ± 35.49 | 1.66E-02 |
| Serum Bilirubin —mg/dL | 5.29 ± 4.19 | 2.37 ± 2.08 | 1.96 ± 1.96 | 3.03E-29 |
| INR | 1.81 ± 0.45 | 1.53 ± 0.31 | 1.42 ± 0.29 | 7.59E-28 |
| Serum Sodium — mEq/L | 134.03 ± 5.77 | 135.72 ± 6.36 | 136.74 ± 4.62 | 9.14E-08 |
| White Blood Cells — x10^3^/mm^3^ | 7.25 ± 4.64 | 5.39 ± 2.67 | 5.57 ± 3.44 | 7.04E-06 |
| ** CRP=C-Reactive Protein, INR= International Normalized Ratio.* | | | | |
| *¶ Counting the hospital admission visit.* |  |  |  |  |

**Table S12. Comparison of the cumulative incidence models at different time points.** Summary of the evaluated goodness-of-fit metrics to compare the cumulative incidence models based on the stratification at hospital admission *vs* at the last reported visit before any event occurrence in the PREDICT cohort.

|  |  | **N=688** | |
| --- | --- | --- | --- |
|  |  | **Hospital Admission** | **"Last visit"** |
| **ACLF** | Log-rank Test | 3e-04 | 1e-11 |
|  | Likelihood ratio | 15.69 | 38.16 |
|  | Concordance | 0.613 | 0.665 |
|  | BIC | 1021.72 | 994.87 |
|  | AIC | 1016.96 | 992.48 |
| **Death** | Log-rank Test | 2e-04 | 1e-09 |
|  | Likelihood ratio | 18.45 | 31.5 |
|  | Concordance | 0.621 | 0.646 |
|  | BIC | 1060.07 | 1042.59 |
|  | AIC | 1055.23 | 1040.18 |
| *BIC= Bayesian Information Criterion, AIC=Akaike Information Criterion* | | | |
