# Supplementary material for: A robust clustering strategy for stratification unveils unique patient subgroups in acutely decompensated cirrhosis
Source: J Transl Med. 2024 Jun 27;22:599. doi: 10.1186/s12967-024-05386-2 (PMC11210156; doi:10.1186/s12967-024-05386-2)
Supplement: Supplementary file 2 — Supplementary Material 2 [file 12967_2024_5386_MOESM2_ESM.pdf]

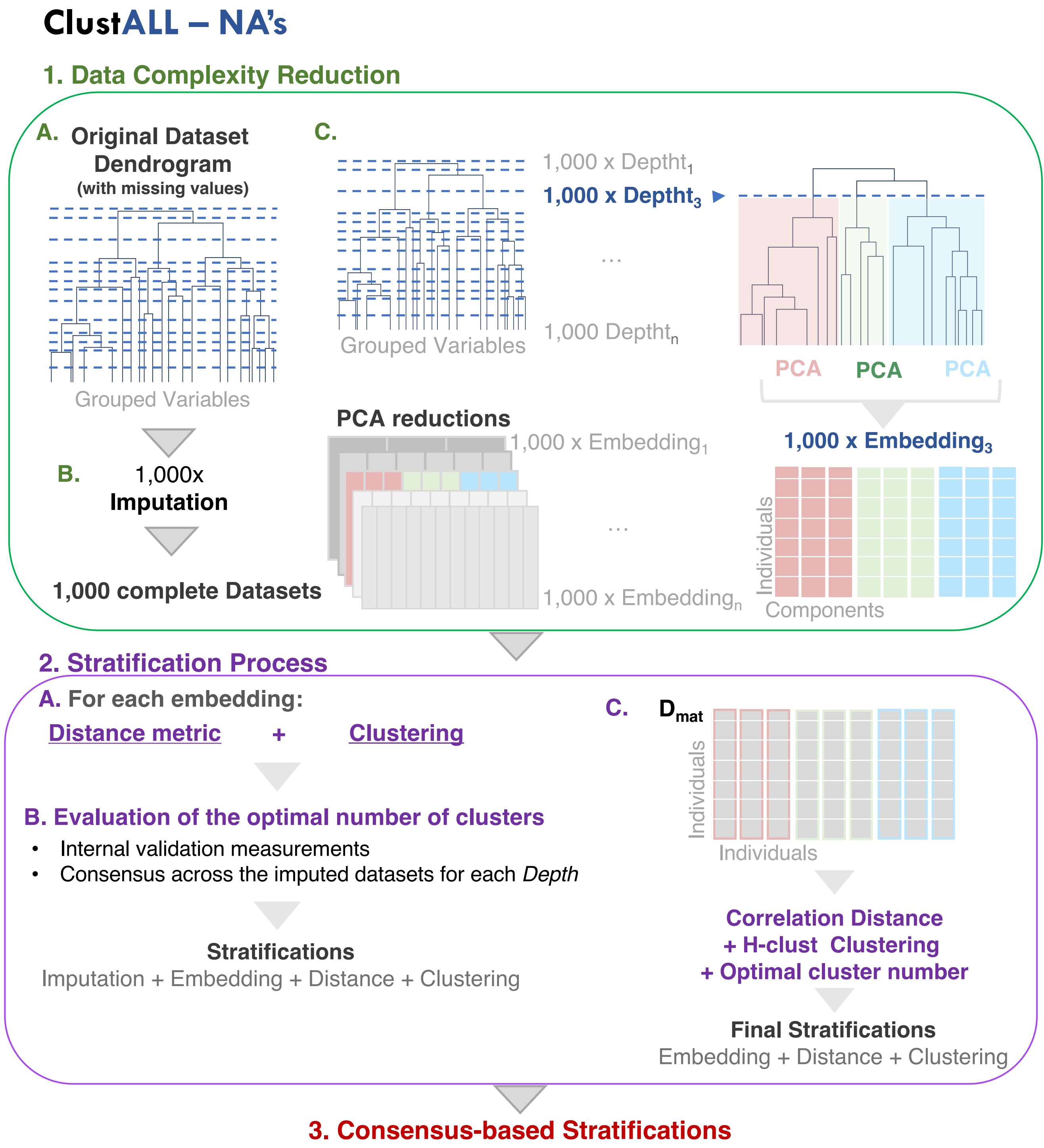

ClustALL – NA's
1. Data Complexity Reduction
A. Original Dataset Dendrogram
(with missing values)
Grouped Variables
B. 1,000x Imputation
1,000 complete Datasets
C.
1,000 x Deptht$_1$
1,000 x Deptht$_3$
...
1,000 Deptht$_n$
Grouped Variables
PCA
PCA
PCA
PCA reductions
1,000 x Embedding$_1$
...
1,000 x Embedding$_n$
1,000 x Embedding$_3$
Individuals
Components
2. Stratification Process
A. For each embedding:
Distance metric + Clustering
B. Evaluation of the optimal number of clusters
• Internal validation measurements
• Consensus across the imputed datasets for each *Depth*
Stratifications
Imputation + Embedding + Distance + Clustering
C.
D_{mat}
Individuals
Individuals
Correlation Distance
+ H-clust Clustering
+ Optimal cluster number
Final Stratifications
Embedding + Distance + Clustering
3. Consensus-based Stratifications
