# Supplementary figures and images for: A robust clustering strategy for stratification unveils unique patient subgroups in acutely decompensated cirrhosis
Source: J Transl Med. 2024 Jun 27;22:599. doi: 10.1186/s12967-024-05386-2 (PMC11210156; doi:10.1186/s12967-024-05386-2)

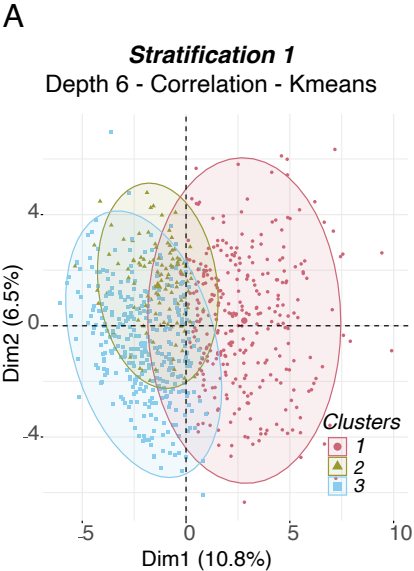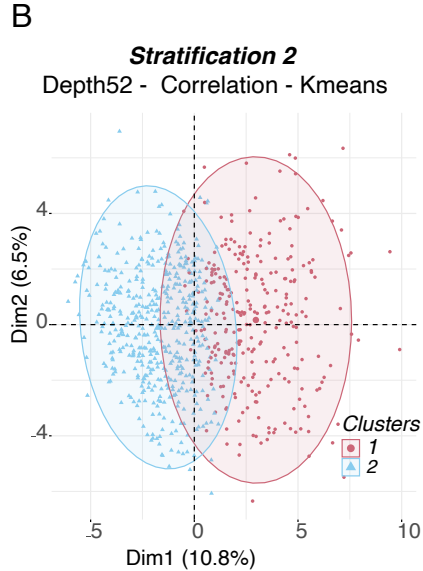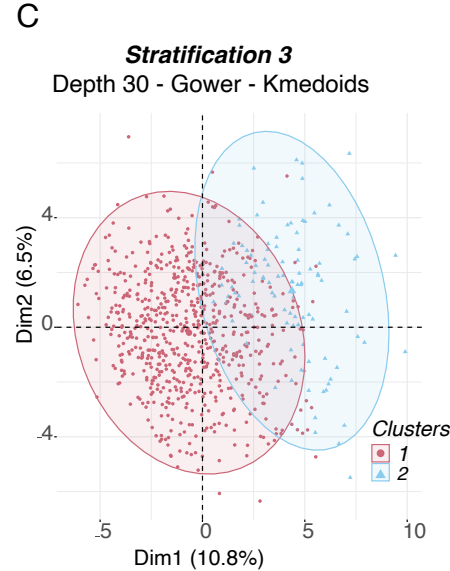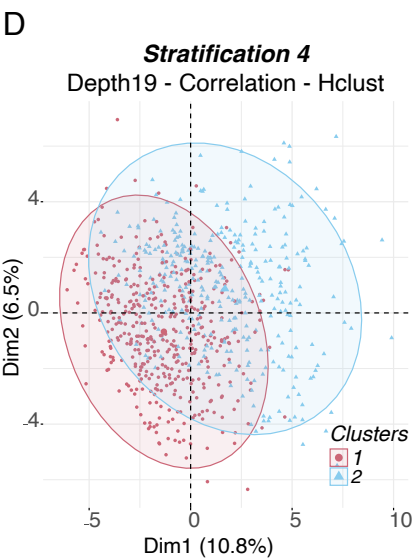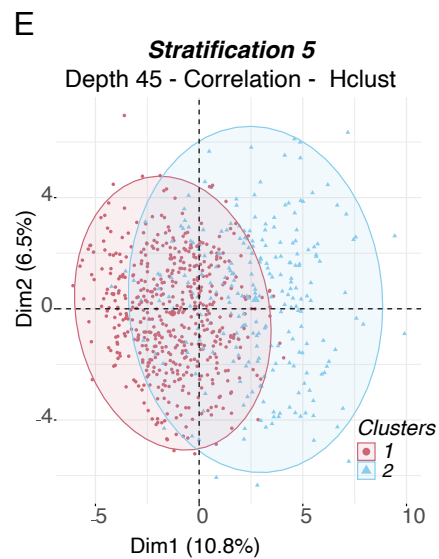

Supplement: Supplementary file 3 — Supplementary Material 3 [file 12967_2024_5386_MOESM3_ESM.pdf]

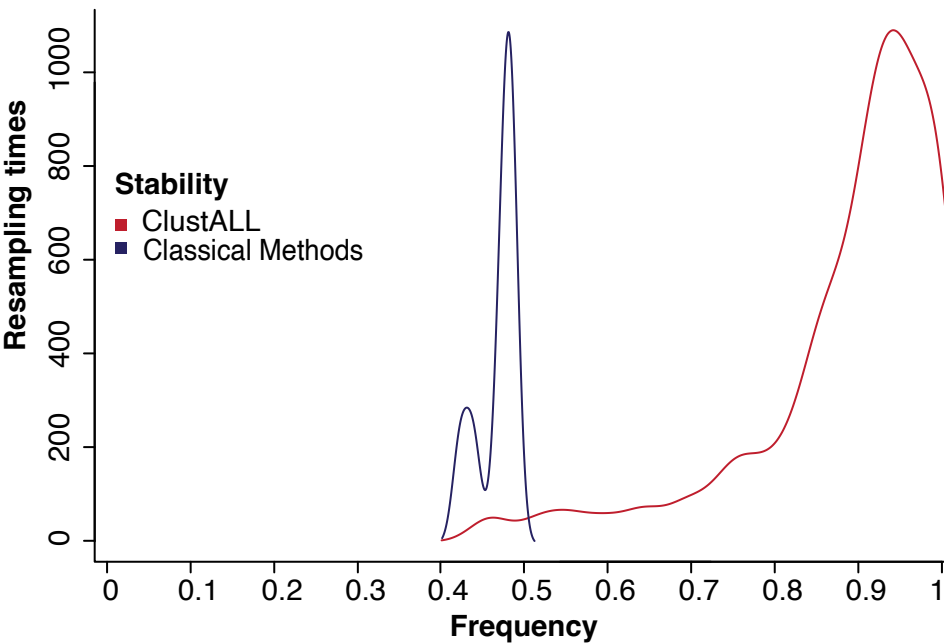

Supplement: Supplementary file 4 — Supplementary Material 4 [file 12967_2024_5386_MOESM4_ESM.pdf]

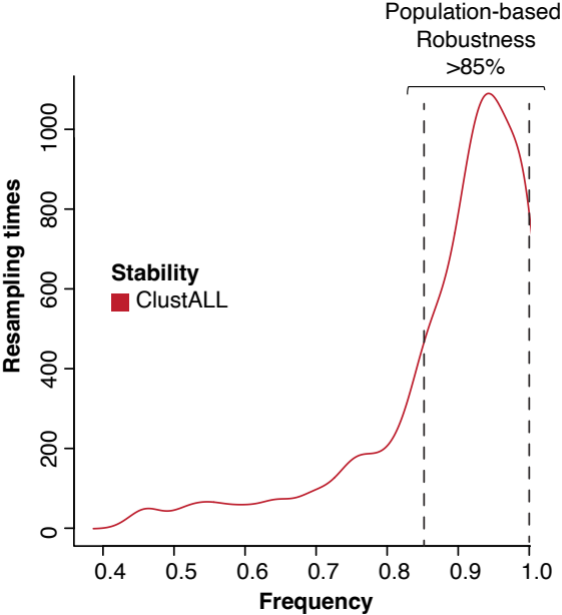

Supplement: Supplementary file 5 — Supplementary Material 5 [file 12967_2024_5386_MOESM5_ESM.pdf]

**Distribution of the window of time between the last reported visit and the reported event (N = 147)**

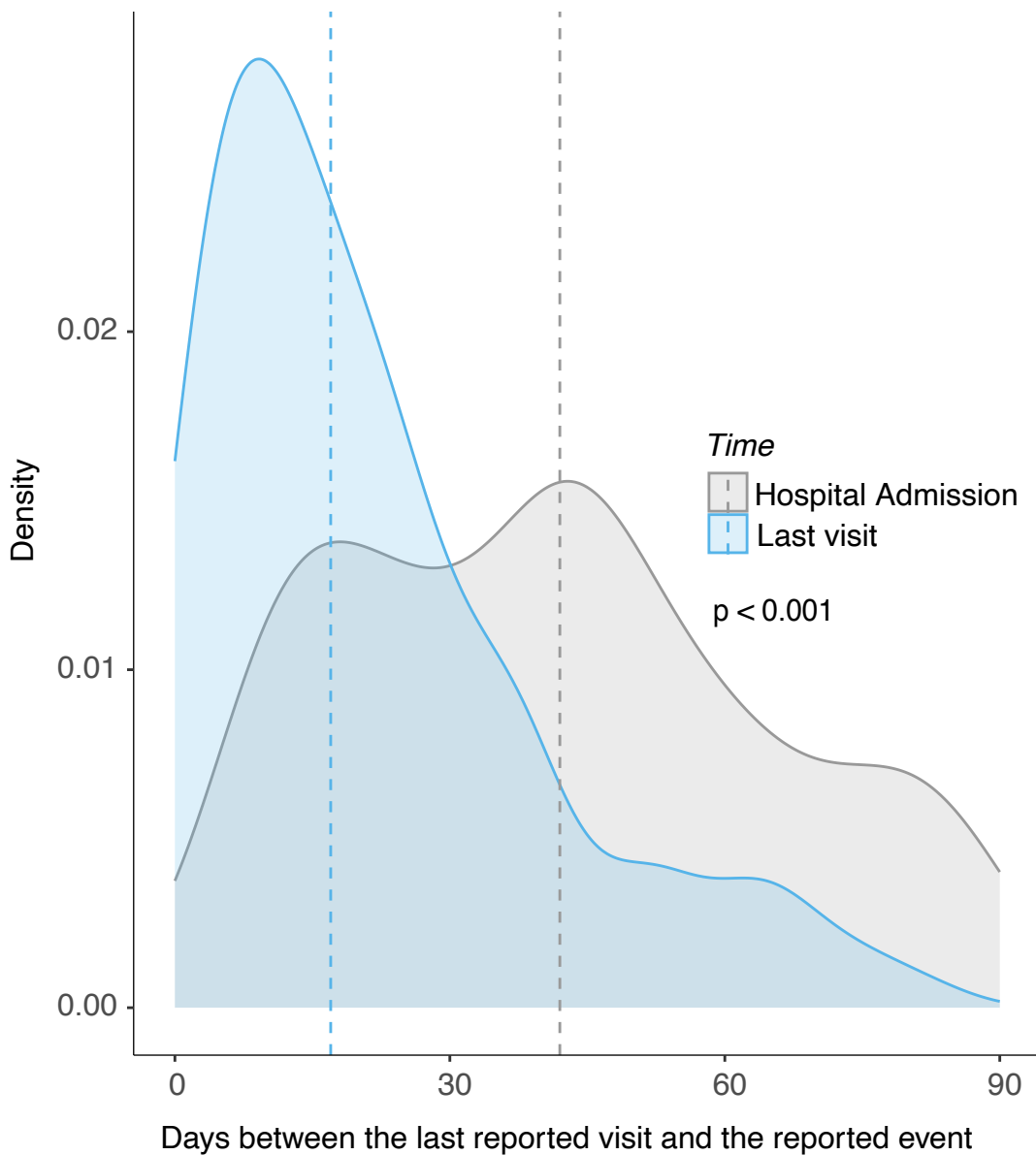

Supplement: Supplementary file 6 — Supplementary Material 6 [file 12967_2024_5386_MOESM6_ESM.pdf]
